# Circulating monocytes associated with anti-PD-1 resistance in human biliary cancer induce T cell paralysis

**DOI:** 10.1016/j.celrep.2022.111384

**Published:** 2022-09-20

**Authors:** Bridget P. Keenan, Elizabeth E. McCarthy, Arielle Ilano, Hai Yang, Li Zhang, Kathryn Allaire, Zenghua Fan, Tony Li, David S. Lee, Yang Sun, Alexander Cheung, Diamond Luong, Hewitt Chang, Brandon Chen, Jaqueline Marquez, Brenna Sheldon, Robin K. Kelley, Chun Jimmie Ye, Lawrence Fong

**Affiliations:** 1 Division of Hematology/Oncology, University of California, San Francisco, San Francisco, CA, USA; 2 Cancer Immunotherapy Program, University of California, San Francisco, San Francisco, CA, USA; 3 Helen Diller Family Comprehensive Cancer Center, University of California, San Francisco, San Francisco, CA 94143, USA; 4 Department of Epidemiology and Biostatistics, University of California, San Francisco, San Francisco, CA, USA; 5 Institute for Human Genetics, University of California, San Francisco, San Francisco, CA, USA; 6 Division of Rheumatology, Department of Medicine, University of California, San Francisco, San Francisco, CA, USA; 7 Department of Microbiology and Immunology, University of California, San Francisco, San Francisco, CA, USA; 8 Department of Genome Sciences, University of Washington, Seattle, WA, USA; 9 Bakar Computational Health Sciences Institute, University of California, San Francisco, San Francisco, CA, USA; 10 Chan Zuckerberg Biohub, San Francisco, CA, USA; 11 J. David Gladstone-UCSF Institute of Genomic Immunology, San Francisco, CA, USA; 12 Parker Institute for Cancer Immunotherapy, San Francisco, CA, USA; 13 Lead contact

## Abstract

Suppressive myeloid cells can contribute to immunotherapy resistance, but their role in response to checkpoint inhibition (CPI) in anti-PD-1 refractory cancers, such as biliary tract cancer (BTC), remains elusive. We use multiplexed single-cell transcriptomic and epitope sequencing to profile greater than 200,000 peripheral blood mononuclear cells from advanced BTC patients (n = 9) and matched healthy donors (n = 8). Following anti-PD-1 treatment, CD14^+^ monocytes expressing high levels of immunosuppressive cytokines and chemotactic molecules (CD14_CTX_) increase in the circulation of patients with BTC tumors that are CPI resistant. CD14_CTX_ can directly suppress CD4^+^ T cells and induce SOCS3 expression in CD4^+^ T cells, rendering them functionally unresponsive. The CD14_CTX_ gene signature associates with worse survival in patients with BTC as well as in other anti-PD-1 refractory cancers. These results demonstrate that monocytes arising after anti-PD-1 treatment can induce T cell paralysis as a distinct mode of tumor-mediated immunosuppression leading to CPI resistance.

## In brief

Keenan et al. report that a monocyte population expressing immunosuppressive chemokines and cytokines (CD14_CTX_) is induced with anti-PD-1 and associates with resistance to treatment in biliary tract cancer. CD14_CTX_ induce immune-paralyzed CD4^+^ T cells, representing an alternative mechanism of resistance in checkpoint inhibitor-refractory cancers.

## Graphical Abstract

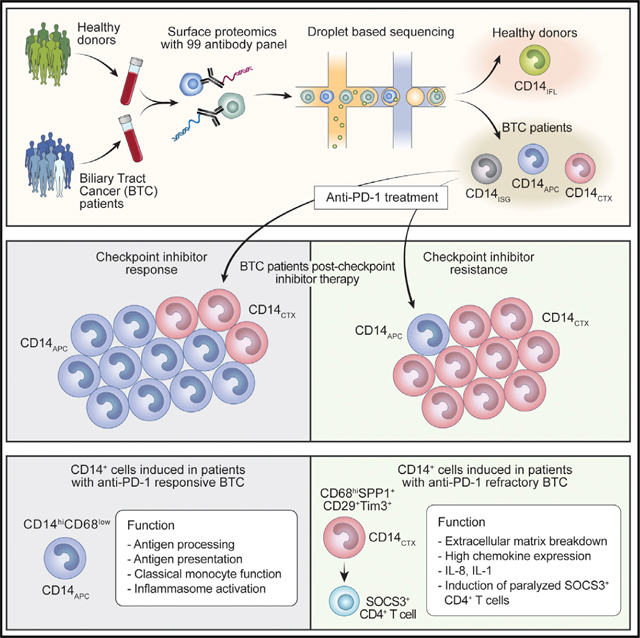

## INTRODUCTION

While immune checkpoint inhibition (CPI) can lead to dramatic clinical responses in specific cancers, many solid malignancies are insensitive to this treatment approach. Advanced biliary tract cancers (BTCs), a family of heterogeneous epithelial cancers including intrahepatic and extrahepatic cholangiocarcinoma and gallbladder cancer, have a poor prognosis and an objective response rate to CPI under 10% (Kim et al., 2020; Piha-Paul et al., 2020). While BTCs have been characterized by an immunosuppressive microenvironment, desmoplastic stroma, and a paucity of tumor-infiltrating effector T cells (Rizvi et al., 2018; Zhou et al., 2019), the mechanisms that underlie primary resistance to CPI are not fully elucidated.

Cells of the myeloid lineage consist of both tumor-promoting and -suppressing subsets that function in inflammation and cancer immunity (Broz and Krummel, 2015; DeNardo et al., 2011; Linde et al., 2018; Ma et al., 2021). While CPI was developed to target T cells and its effects on various subsets have been well-documented, its effects on myeloid cells are not as well understood despite associations to altered frequency and activation states of myeloid cells (Hartley et al., 2018). For example, an increased frequency of circulating CD14^+^CD16^−^HLA-DR^hi^ monocytes prior to treatment, along with a decreased frequency of T cells, correlate with survival and response to anti-PD-1 in melanoma patients (Krieg et al., 2018). Further, PD-1 signaling can polarize macrophages to an M2 phenotype, lead to defects in phagocytosis, and impair effective anti-tumor immunity (Diskin et al., 2020; Gordon et al., 2017; Strauss et al., 2020).

Here, we used sample multiplexed single-cell cellular indexing of transcriptomes and epitopes (CITE-seq) to dissect the Cell compositional, transcriptomic, and surface proteomic responses of circulating immune cells from BTC patients to CPI. We found the composition of immune cells from BTC patients differed drastically from healthy donors, and that further changes were induced in patients’ circulation following anti-PD-1 treatment, particularly in CD14^+^ monocytes. Monocytes with canonical monocytic features are associated with response, while those expressing specific immunosuppressive markers are associated with insensitivity to CPI and can directly suppress CD4^+^ T cells. By analyzing surface protein and expression profiles across individuals, we further showed associations of immunosuppressive monocytes with the frequency of *SOCS3*^*+*^ CD4^+^ T cells in BTC patients. In co-culture experiments, these immunosuppressive monocytes were able to render resting CD4^+^ T cells unresponsive, suggesting multiple mechanisms of inhibition.

## RESULTS

### Multiplexed CITE-seq identifies altered circulating immune cell composition in BTC patients compared with healthy individuals

**Figure 1.**
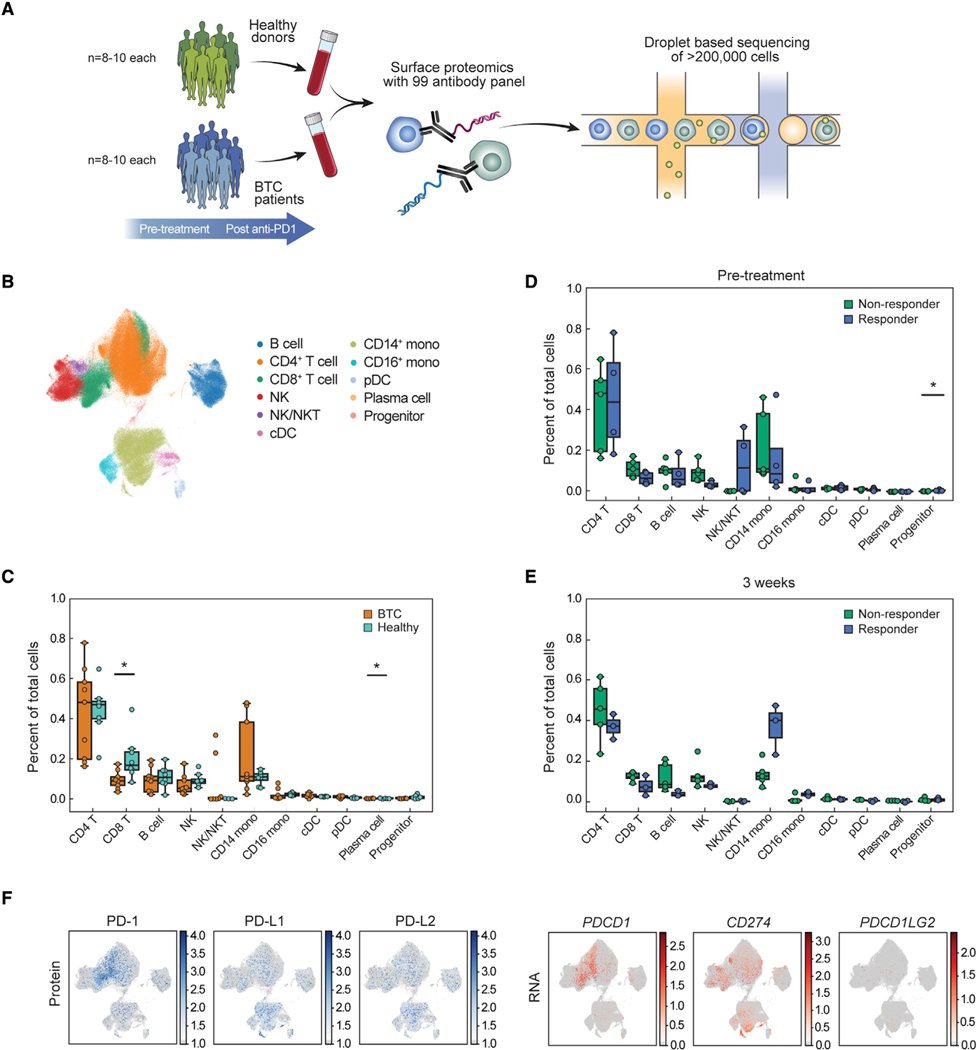
Analysis of circulating immune cells within healthy individuals and BTC patients (A) Schematic of experimental design. (B) Uniform manifold approximation and projection (UMAP) plot of all cells from BTC patient and healthy donor blood samples colored by cell type. NK/NKT cluster contains T cells, NK T cells, and NK cells; cDC = conventional dendritic cells; mono = monocytes; pDC = plasmacytoid dendritic cells. (C–E) Percent of each cell type out of total immune cells in BTC patients (prior to treatment, n = 9) and healthy donors (n = 8) (C) and in responders (n = 4) and non-responders (n = 5) prior to treatment (D), and 3 weeks following anti-PD-1 (E). * denotes significance (adjusted p < 0.05). Boxes denote inter-quartile range (IQR), while bars denote 25% – 1.5 3 IQR and 75% + 1.5xIQR. (F) UMAP of all immune cells colored by protein and RNA expression for PD-1 (*PDCD1*), PD-L1 (*CD274*), and PD-L2 (*PDCD1LG2*).

We used multiplexed CITE-seq to profile peripheral blood mononuclear cells (PBMC) obtained from BTC patients (n = 9) before, 1 week after, and 3 weeks after anti-PD-1 treatment as well as from gender- and age-matched healthy donors (n = 8) (Table S1 and Figure 1A). By analyzing over 230,000 cells, we identified the canonical circulating myeloid and lymphoid cell types including B cells, CD4^+^ and CD8^+^ T cells, NK cells, NK T cells, plasmacytoid and conventional dendritic cells (pDC, cDC), CD14^+^ and CD16^+^ monocytes, plasma cells, and a small immune progenitor cell population visualized using uniform manifold approximation and projection (UMAP) (Figures 1B and S1A–S1G and Table S2). When comparing pre-treatment BTC patients’ circulating immune cells with healthy donors, we found decreased frequencies of CD8^+^ and plasma cells in BTC patients (Figure 1C and Table S3). We next examined whether there were differences in circulating immune cells in BTC patients when analyzed by their clinical outcome to treatment. With the exception of the small progenitor cell population, there were no significant differences in frequencies of broad immune cell types in patients whose tumors responded to anti-PD-1 (responder) or were insensitive (non-responder) prior to or following CPI (Figures 1D, 1E, S1H, and Table S3). These findings differ from those reported in melanoma patients, in which an increased frequency of circulating CD14^+^ monocytes was observed in patients whose tumor responded to immunotherapy prior to treatment (Krieg et al., 2018). While T cells are thought to be the major targets of anti- PD-1 therapy, we found that CD14^+^ monocytes express high levels of PD-L1 and PD-L2 transcript and protein expression and PD-1 surface protein expression before CPI treatment (Figure 1F). These results are consistent with previously published reports (Diskin et al., 2020; Gordon et al., 2017; Strauss et al., 2020) and suggest that anti-PD-1 may also act on myeloid cells.

### BTC patients harbor distinct populations of circulating myeloid cells

**Figure 2.**
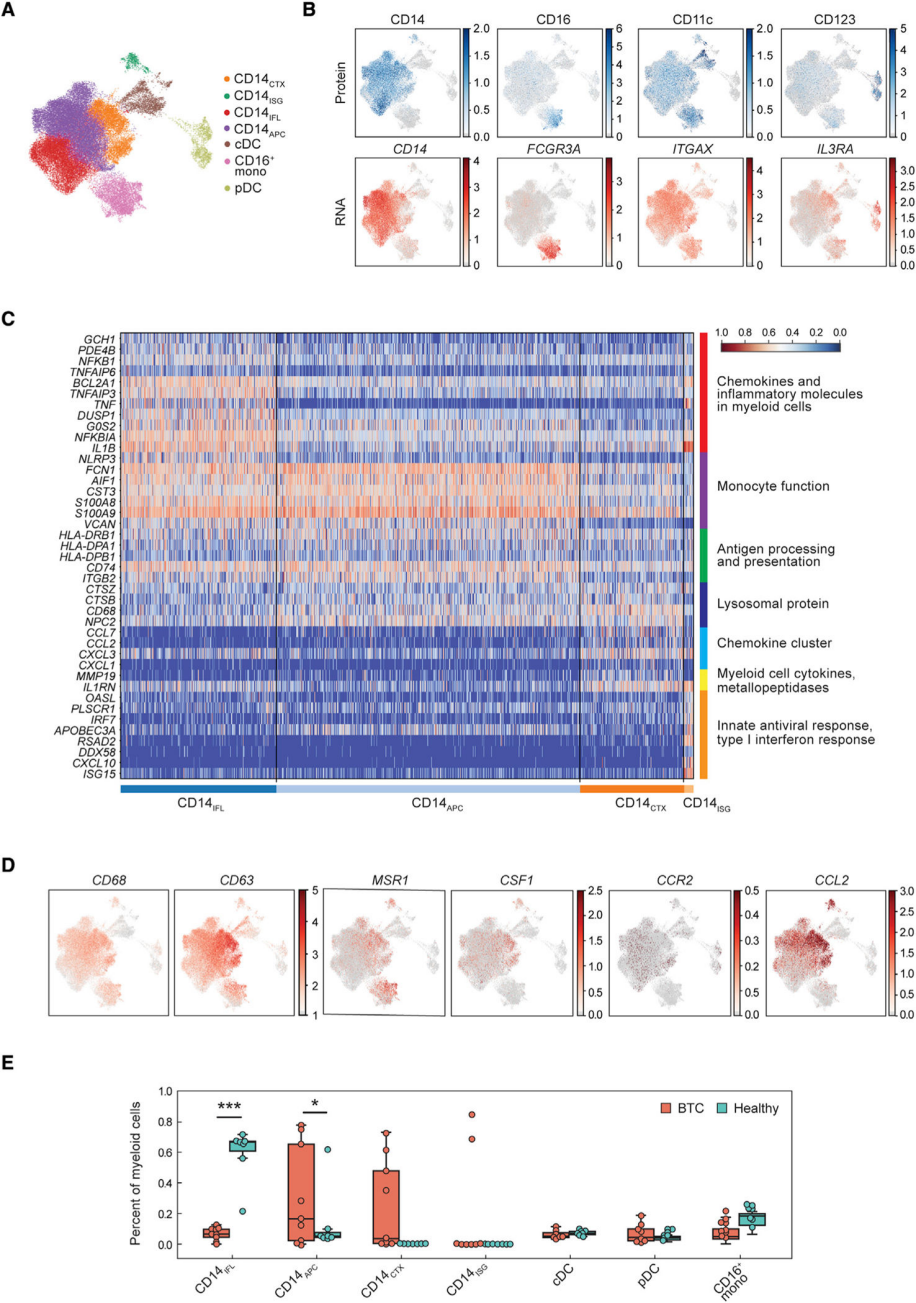
Circulating myeloid populations within BTC patients and healthy responders (A) UMAP colored by myeloid cell subtype. cDC = conventional dendritic cells; mono = monocytes; pDC = plasmacytoid dendritic cells. (B) UMAP of myeloid cells showing expression of each protein or RNA molecule used to annotate myeloid subtypes. (C) Heatmap with expression of genes in the top enriched pathways (right labels) for each monocyte subtype. (D) UMAP of RNA expression of the indicated gene across all myeloid cells. (E) Percent of each cell subtype out of total myeloid cells in BTC patients (prior to treatment, n = 9) and healthy donors (n = 8). * denotes significance (adjusted p < 0.05); *** denotes adjusted p value < 0.001. Boxes denote inter-quartile range (IQR), while bars denote 25% – 1.5xIQR and 75% + 1.5xIQR.

To focus further on the myeloid compartment, we re-clustered on the monocytes and dendritic cells and identified seven sub-populations annotated using a combination of protein and RNA markers (Figures 2A and 2B). These included conventional dendritic cells (cDC), plasmacytoid dendritic cells (pDC), CD16^+^ monocytes, and four sub-populations of CD14^+^ monocytes. We used gene ontology enrichment analysis of upregulated genes (Ashburner et al., 2000; The Gene Ontology, 2019) to annotate the four CD14^+^ monocyte sub-populations by canonical immune-specific pathways (Li et al., 2014) (Figure 2C and Tables S4): (1) CD14_IFL_ myeloid cells were enriched for pathways related to inflammation (e.g., pro-inflammatory cytokines and chemokines, NFkB signaling, and inflammasome function); (2) CD14_APC_ cells were enriched in monocyte differentiation and function and antigen processing and presentation; (3) CD14_ISG_ represented a smaller population of CD14^low^ monocytes with upregulated interferon response genes (ISG) and innate immune signaling; and (4) CD14_CTX_ cells were enriched for chemotaxis molecules and suppressive cytokines. CD14_CTX_ also have increased expression of macrophage-associated genes *CD63*, *CD68*, *MSR1*, *CFS1*, *CCL2*, and *CCR2* and lower expression of *CD14* (Figures 2B and 2D). These findings suggest that CD14_APC_, CD14_IFL_, and CD14_ISG_ are canonical CD14^+^ monocytes, while CD14_CTX_ may exist on the spectrum of monocytes-macrophages (Betjes et al., 1991; Iqbal et al., 2014). The distribution of CD14^+^ sub-populations varied between BTC and healthy donors with quantitative differences detected for several populations, despite there not being an apparent difference when comparing total CD14^+^ monocyte frequencies overall (Figure 2E and Table S3). BTC patients prior to treatment had an abundance of the different monocyte sub-populations including CD14_CTX_ and CD14_ISG_, and an increased frequency of CD14_APC_ and a decreased frequency of CD14_IFL_ in comparison with healthy individuals. Healthy donors had a more uniform composition of monocyte sub-populations (predominately CD14_IFL_) (Figure 2E).

### Myeloid subpopulation frequencies and gene signatures differ by clinical outcome

**Figure 3.**
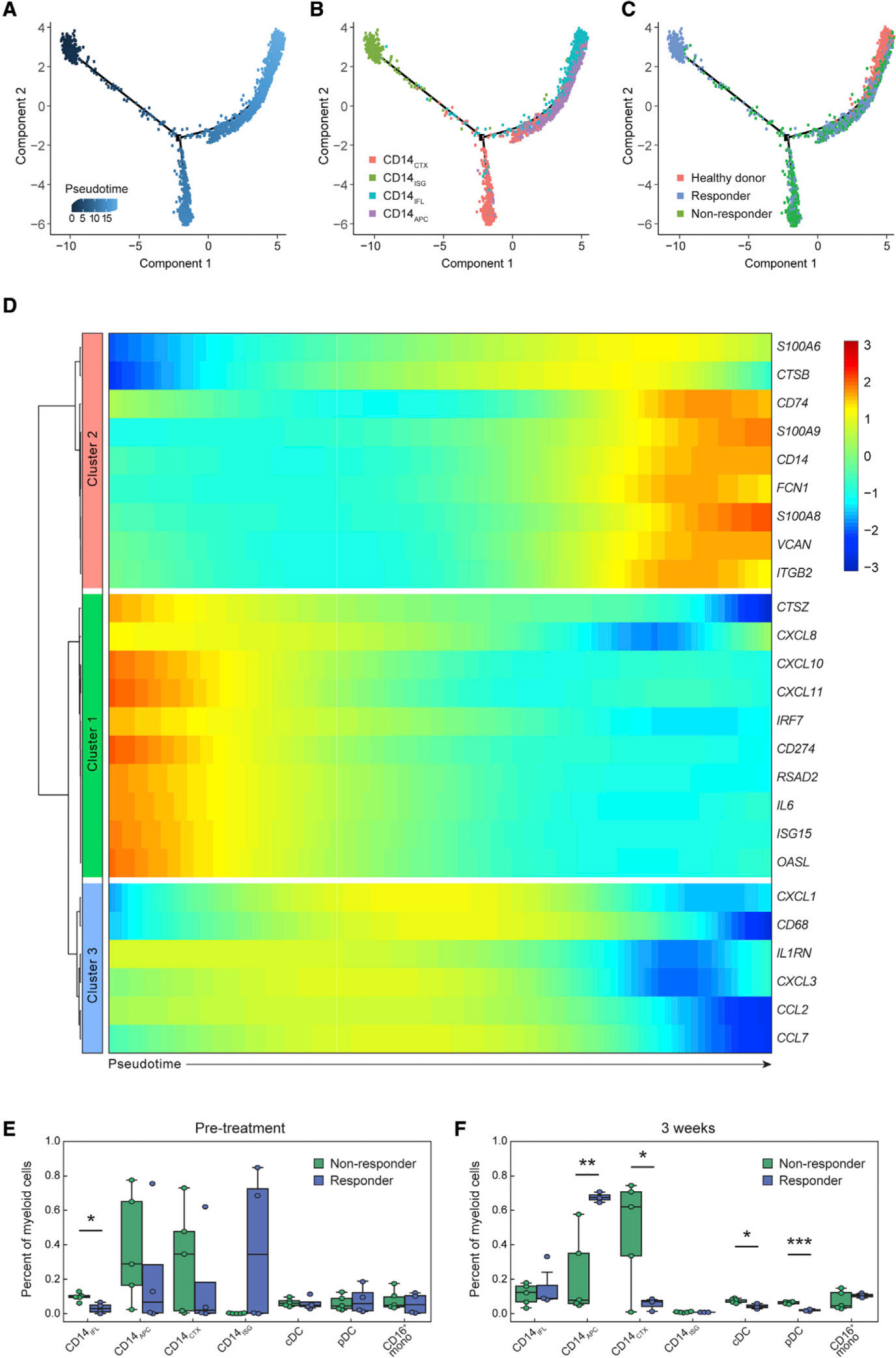
Monocyte subtypes associated with anti-PD-1 response (A–C) Trajectory analysis of monocyte subtypes from BTC patients and healthy donors. Cells are ordered in latent time (A) with monocyte subtype (B) or response status (C) overlaid. (D) Heatmap of differentially expressed genes, arranged by clusters of patterns of gene expression across latent time (direction shown by arrow). (E and F) Percent of each cell subtype out of total myeloid cells in BTC responders (n = 4) and non-responders (n = 5) prior to treatment (E) and 3 weeks following anti-PD-1 (F). * denotes significance (adjusted p < 0.05); ** denotes adjusted p value < 0.005; *** denotes adjusted p value < 0.001. Boxes denote inter-quartile range (IQR) while bars denote 25% – 1.5xIQR and 75% + 1.5xIQR.

To examine whether the circulating monocyte sub-populations may represent states of monocyte-macrophage differentiation, we used trajectory analysis (Trapnell et al., 2014) to order the four CD14^+^ monocyte sub-populations along latent time (Figures 3A–3C). Genes differentially expressed along latent time overlapped with top differentially expressed genes in these populations and organized into three modules, distinct but related to the monocyte cell clusters (Figure 3D). Module 2 genes increase over latent time and include markers of monocytic lineage (*CD14*, *VCAN*, *S100A8*, *S100A9*, *CD74*), whereas modules 1 and 3 decrease over latent time and include ISGs (*RSAD2*, *ISG15*, *IRF7*), PD-L1 (*CD274*), and immunosuppressive cytokines (*CXCL8*, *CXCL10*, *CXCL11*, *IL6*) (Figure 3D and Table S5). Furthermore, monocytes from BTC patients at baseline and 1 week were present across latent time in both responders and non-responders. However, by week 3, monocytes from responders were mainly found alongside the CD14^+^ monocytes from healthy donors (Figures 3C and S2A). Noting differences between CD14^+^ sub-populations in this analysis, we assessed the composition of all myeloid populations by response category (Figures 3E, 3F, and S2B). The small population of CD14_ISG_ was derived from the circulation of two of the four responders at baseline (Figure 3E). There were few other significant differences pre-treatment or at 1 week post-treatment between responders and non-responders (Figures 3E and S2B). However, by 3 weeks post-anti-PD-1, responders had a markedly higher frequency of CD14_APC_, whereas non-responders had an increased frequency of CD14_CTX_, pDC, and cDC (Figure 3F).

### CD14_CTX_ express a program of immunosuppressive chemokines and cytokines

**Figure 4.**
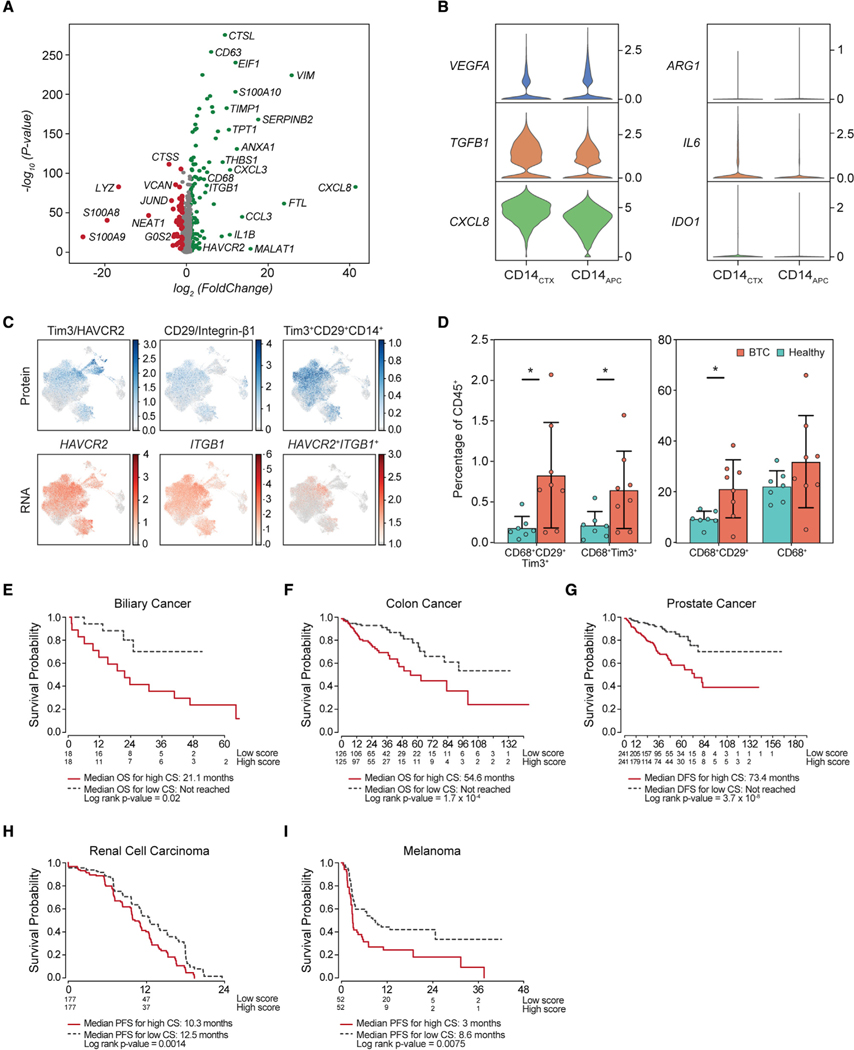
Monocyte gene signatures associated with poor prognosis in CPI-insensitive cancer types (A) Volcano plot of log_2_(fold change) and –log_10_(p value) showing differently expressed genes between CD14_CTX_ and CD14_APC_. (B) Expression of suppressive chemokines and cytokines associated with MDSC and M2 macrophages is shown for CD14_CTX_ and CD14_APC_. (C) Protein (top panel) and RNA (bottom panel) expression overlaid on UMAP of myeloid cells for *HAVCR2* (Tim3) and *ITGB1* (CD29, integrin-α1) and for the combination of both genes/proteins. (D) Bar plots of each myeloid population gated on CD68 and calculated as percentage of total CD45^+^ circulating immune cells as analyzed by flow cytometry of peripheral blood samples from healthy donors (‘‘Healthy,’’ n = 7) or BTC patients (n = 8). * = p < 0.05, error bars denote standard deviation. (E–G) Kaplan-Meier curve of overall survival for cholangiocarcinoma (E) and colon cancer (F), and disease-free survival for prostate cancer (G) cases in the TCGA dataset by high (red line: median expression greater than composite score [CS]) or low (dashed line: median expression lower than CS) expression of the CD14_CTX_ gene signature. (H and I) Kaplan-Meier curve of progression-free survival for renal cell carcinoma (H) and melanoma (I) patients treated with PD-1 blockade inhibition by high (red) or low (black) expression of the CD14_CTX_ gene signature. For (E)–(I), the y axis is in months, and the numbers below the plots denote number of individuals at risk. NR = not reached, CI = confidence interval, OS = overall survival, DFS = disease-free survival, PFS = progression-free survival.

We next used MAST (Finak et al., 2015) to compare CD14_CTX_ to CD14_APC_ cells, the two dominant populations in BTC patients’ circulation following CPI. CD14_CTX_ had increased expression of several tumor-associated macrophage (TAM)- and/or myeloid-derived suppressor cell (MDSC)-related cytokines (Mantovani et al., 2017; Ostrand-Rosenberg and Fenselau, 2018), including *IL6*, *TGFB1*, and *CXCL8* (Figures 4A and 4B). However, CD14_CTX_ lack expression of other MDSC-associated genes including *ARG1*, *VEGFA*, and *IDO1* (Figure 4B and Table S6). Although antigen processing and presentation pathways were enriched in both monocyte sub-populations (Figures S3A and S3B), the individual genes and pathways differed. CD14_CTX_ expressed COX2 (*PTSG2*) and HLA molecules (Figure S3B), while CD14_APC_ expressed genes related to monocyte surface phenotype (*S100A8*, *S100A9*, *CD14*, *FCN1*) and function (i.e., the inflammasome-related gene, *NLRP3*) (Figures 4A and S3A and Table S6). CD14_CTX_ also expressed a distinct set of chemokines involved in the recruitment of CCR2^+^ inflammatory monocytes, a population associated with poor outcomes in cancer patients (*CCL2*, *CCL7*) (Geissmann et al., 2003; Sanford et al., 2013), recruitment of neutrophils (*CXCL1*, *CXCL2*, *CXCL3*) (Mollica Poeta et al., 2019; Sokol and Luster, 2015), and associated with T cell exhaustion (*CCL20*) (Kfoury et al., 2021), pro-inflammatory cytokines (*IL1A*, *IL1B*), as well as molecules associated with cell migration and extracellular matrix digestion (*TIMP1*, *CTSB*, *CTSZ)* (Akkari et al., 2014; Porter et al., 2013; Roeb et al., 2005) (Figures 4A and S3B).

To enable further *in vitro* functional characterization, we leveraged the surface protein abundance data from CITE-seq to identify markers that can distinguish CD14_CTX_ from other monocyte sub-populations. First, we used COMET (Delaney et al., 2019) to identify two highly expressed surface markers in CD14_CTX_: Tim3 (*HAVCR2*), an immune checkpoint on T cells that is also expressed by dendritic cells and M2 macrophages (Ocana-Guzman et al., 2016), and CD29 (*ITGB1*), an integrin that can mediate chemotaxis (Shang et al., 1998) and is upregulated in macrophages compared with other myeloid cells (Ammon et al., 2000). We confirmed that Tim3 and CD29 are highly expressed by CD14_CTX_ at the RNA and protein levels and that the combination specifically distinguishes CD14_CTX_ from other sub-populations (Figure 4C). Using flow cytometry, we demonstrated that BTC patients had an increased frequency of CD29^+^Tim3^+^CD68^+^ cells as well as Tim3^+^CD68^+^ and CD29^+^CD68^+^ cells compared with healthy donors, with similar findings shown for CD14-gated cells (Figures 4D and S3C–S3F). Enrichment of CD29^+^ and Tim3^+^ monocytes was specific to BTC patients, while the frequency of total CD14^+^ or CD68^+^ myeloid cells did not differ significantly between healthy individuals and BTC patients (Figures 4D and S3F). To validate these findings, flow cytometry was performed on PBMC from additional BTC patients (n = 16), confirming an increased frequency of CD29^+^Tim3^+^ monocytes (gated by either CD68 or CD14) (Figure S3G).

### CD14_CTX_ gene signature correlates with SPP1^+^ TAMs in the tumor microenvironment and is associated with poor prognosis in other CPI-insensitive tumors

Because CD14_CTX_ express chemokine receptors that might facilitate migration into the tissues, we next examined the relationship between circulating and intra-tumoral myeloid states in BTC. We performed scRNA-seq on primary cholangiocarcinoma tumors (n = 4) obtained from standard of care resections (Table S7) and recovered a total of 10,913 myeloid cells. Tissue-associated myeloid cells consisted of dendritic cells (DC), neutrophils (Neut), two populations of macrophages characterized by either high *APOE* or *SPP1* expression (Mac_APOE_, Mac_SPP1_), CD14^+^ monocytes (CD14^+^ mono), CD16^+^ monocytes (CD16^+^ mono), and intermediate *CD14*^+^*CD16*^+^ monocytes (CD14^+^CD16^+^ mono) (Figures S4A and S4B). Among the tissue-associated myeloid populations, the expression profile of CD14_CTX_ was most correlated with Mac_SPP1_, exemplified by the shared expression of differentially expressed CD14_CTX_ genes including *HAVCR2* and *ITGB1* (Figures S4C and S4D). Notably, two genes that differ in expression between Mac_SPP1_ and CD14_CTX_ are related to chemotaxis and extravasation (*SERPINB2* [Schroder et al., 2019], *TIMP1* [Roeb et al., 2005]), suggesting a transition in macrophages that have already migrated to the tumor microenvironment. As an alternate approach to assess the relationship between the circulating and tissue-associated myeloid populations, we co-clustered myeloid cells from circulation with those from the tumors, following batch correction. We found that the intra-tumoral Mac_SPP1_ cluster with the circulating CD14_CTX_ (Figure S4E). An alternative approach using partition-based graph abstraction (Wolf et al., 2019) also found close connectivity between CD14_CTX_ and SPP1^+^ TAM clusters (Figure S4E). We also found evidence of *SPP1*^+^*HAVCR2*^+^*CD68*^+^ myeloid cells within biliary tumor tissue from on-treatment biopsies by combined *in situ* hybridization and immunofluorescence, further suggesting the existence of a TAM population in BTC analogous to CD14_CTX_ (Figure S4F).

To test whether CD14_CTX_ gene signature may be prognostically relevant, we applied the CD14_CTX_ gene signature to the TCGA cholangiocarcinoma dataset (median overall survival = 40.13 months, n = 36) (Farshidfar et al., 2017). High expression of the CD14_CTX_ gene signature was indeed associated with a significantly worse overall survival (median survival = 21.1 months versus not reached, p value = 0.02) (Figure 4E). We then turned to two other prototypical CPI-insensitive cancers: colorectal (O’Neil et al., 2017) and prostate cancer (Antonarakis et al., 2020). We found the CD14_CTX_ gene signature was correlated with worse prognosis in both of these diseases as well. In colon cancer, a high CD14_CTX_ expression score (Table S6) correlated with overall survival of 54.6 months versus not reached for patients with a lower score (p = 1.7 × 10^−4^, n = 251, Figure 4F). In prostate cancer, a higher CD14_CTX_ gene signature expression score correlated with lower disease-free survival (DFS) (73.4 months versus not reached, p = 3.7 × 10^−8^, n = 482) (Figure 4G) (Cancer Genome Atlas, 2012; Cancer Genome Atlas Research, 2015). We next investigated whether the CD14_CTX_ gene signature correlated with outcomes in immunotherapy-treated patients. First, in baseline tumor biopsies from advanced renal cell carcinoma patients (n = 886; NCT02684006) (Choueiri et al., 2020; Motzer et al., 2020), the CD14_CTX_ gene signature correlated with worse progression-free survival in patients treated with avelumab (anti-PD-L1)-based treatment compared with patients whose tumors had a lower CD14_CTX_ gene signature (10.3 versus 12.5 months) (Figure 4H). Secondly, in metastatic melanoma, the CD14_CTX_ gene signature in baseline biopsies was also correlated with worse progression-free survival (3 versus 8.6 months) with pembrolizumab (anti-PD-1) treatment: (n = 112) (Jerby-Arnon et al., 2018) (Figure 4I).

### CD14_CTX_ frequency correlates with SOCS3^+^CD4^+^ T cell frequency

**Figure 5.**
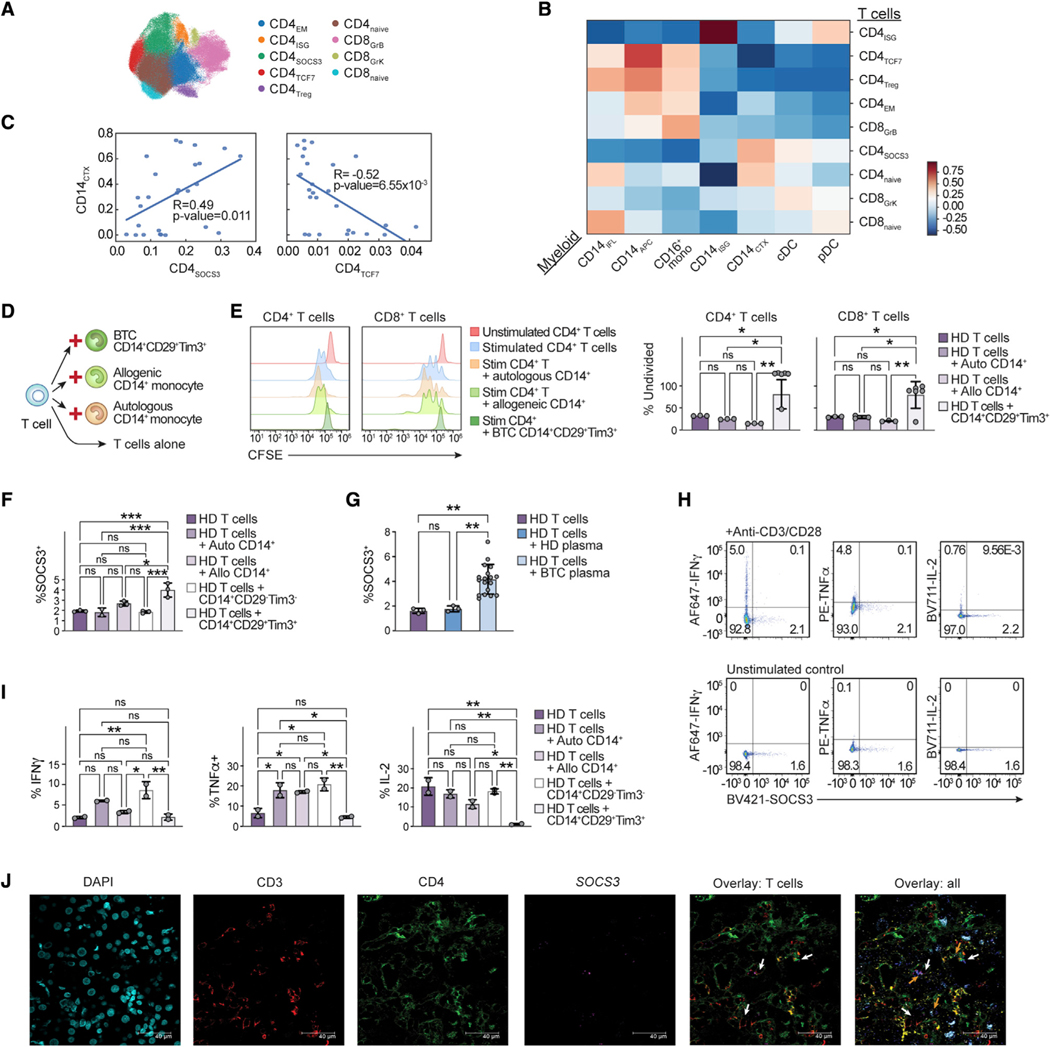
CD14_CTX_ are associated with *SOCS3*^+^CD4^+^ T cells and can induce CD4^+^ T cell suppression (A) UMAP of all T cells in healthy donors and BTC patients colored by cell annotations. (B) Heatmap of Pearson correlation coefficients for cell type frequencies for myeloid and T cell subtypes. (C) The frequency of the specified cell type out of total myeloid or T cells was calculated and then correlated as shown in each plot. Each dot corresponds to an individual patient sample. (D) Schematic of co-culture conditions. The monocyte populations indicated were cultured with healthy T cells for 6 days and re-stimulated with anti-CD3/CD28 beads for 3 days prior to harvest. (E) CFSE staining is shown for representative CD4^+^ and CD8^+^ populations (left panel). Data are summarized in bar plots as the percentage of CD4^+^ and CD8^+^ T cells that remain undivided following re-stimulation in each co-culture condition (right panel, n = 3–6 wells per condition). Stim = stimulated. (F) Flow cytometry assessment for percentage SOCS3^+^ out of healthy donor (HD) CD4^+^ T cells co-cultured with the indicated monocyte population (n = 2–3 wells per condition). (G) Flow cytometry assessment for percentage SOCS3^+^ out of HD CD4^+^ T cells alone (n = 3 replicates) or co-cultured with plasma from HD (n = 3) or BTC patients (n = 18).

Next, we defined T cell sub-populations present in healthy donors and BTC patients by re-clustering on the CD4^+^ and CD8^+^ T cells (Figure 5A). Using both transcript and protein markers, we identified nine unique clusters of T cells: 6 CD4^+^ T cell clusters including naive and effector memory (CD4_naive_, CD4_EM_), *FOXP3*^*+*^ regulatory (CD4_Treg_), and cells characterized by high expression of either *TCF7*, *SOCS3*, or ISG (CD4_TCF7_, CD4_SOCS3_, CD4_ISG_), and three clusters of CD8^+^ T cells including naive (CD8_naive_) and effectors expressing either predominantly *GZMB/GZMH* or *GZMK* (CD8_GrB_, CD8_GrK_) (Figures S5A–S5C). We hypothesized that myeloid-T cell interactions could be involved in CPI insensitivity and examined for frequency association between myeloid cell and T cell sub-populations (Figure 5B). Strikingly, we found that in BTC patients, the frequency of CD14_CTX_ were positively correlated with the frequency of CD4_SOCS3_ (R = 0.49, p value = 0.011) and negatively correlated with CD4_TCF7_ frequency (R = −0.52, p value = 6.55 × 10^−3^) (Figure 5C), whereas the frequency of CD14_APC_ was positively correlated with the frequency of CD4_TCF7_ (R = 0.75, p value = 8.72 × 10^−6^) and not correlated with the frequency of CD4_SOCS3_ (R = −0.32, p value = 0.11) (Figure S5D). The positive correlation of CD4_TCF7_ with CD14_APC_ and negative correlation with CD14_CTX_ in BTC patients is intriguing because *TCF7* expression within CD4^+^ T cells is associated with the capability to self-renew (Nish et al., 2017).

### CD14_CTX_ are capable of inducing dysfunction in CD4^+^ T cells

Given the immunosuppressive gene signature of CD14_CTX_ and their correlation in frequency with CD4^+^ T cells expressing *SOCS3*, a negative regulator of cytokine signaling that has been associated with T cell dysfunction (Croker et al., 2003; Jiang et al., 2017), we investigated the capacity of CD14_CTX_ monocytes to alter the proliferation and function of CD4^+^ T cells. Using the markers we identified from CITE-seq analysis and validated by flow cytometry (Tim3, CD29, CD14), we used FACS (fluorescence-activated cell sorting) to isolate CD14_CTX_ from BTC patients’ PBMCs and co-cultured them with healthy donor CD4^+^ T cells (Figures 5D, S6A, and S6B). Compared with autologous or allogeneic healthy donor CD14^+^ monocytes, CD14_CTX_ cells isolated from BTC patients’ circulation could suppress the proliferation of CD4^+^ T cells (Figure 5E). CD29^+^Tim3^+^ CD14_CTX_ cells could also suppress proliferation of CD8^+^ T cells (Figure 5E). Further, we found that CD14_CTX_ could induce SOCS3 expression in sorted resting CD4^+^ T cells compared with healthy donor monocytes (both from the same [autologous] or a different [allogeneic] healthy donor) and to CD29^–^Tim3^−^CD14^+^ cells from BTC patients (non-CD14_CTX_ monocytes) consistent with the association between the frequency of CD14_CTX_ with CD4_SOCS3_ (Figure 5F). To examine whether these effects could be due to a soluble factor, we cultured resting CD4^+^ T cells from healthy donors with plasma from either healthy plasma donors or from BTC patients. We found that plasma from BTC patients could induce SOCS3 in CD4^+^ T cells (Figure 5G). As SOCS3 expression is associated with ‘‘immune paralysis’’ in CD4^+^ T cells in the setting of cytokine exposure (Sckisel et al., 2015), we assessed the functional capacity of SOCS3^+^CD4^+^ T induced by BTC-derived CD14_CTX_ monocytes. While SOCS3^−^CD4^+^ T cells from BTC patients retained the ability to produce IFNγ, TNFα, and IL2, SOCS3^+^CD4^+^ T cells failed to produce these cytokines in response to stimulation (Figure 5H). We demonstrated that CD4^+^ T cells co-cultured with CD29^+^Tim3^+^CD14^+^ cells secreted less TNFα and IL-2 compared with CD29^–^Tim3^−^CD14^+^ or healthy donor monocytes and IFNγ compared with CD29^–^Tim3^−^CD14^+^ monocytes (Figure 5I). Lastly, we examined whether CD14_CTX_ and CD4_SOCS3_ interact within the tumor microenvironment since we could also identify a population of *SOCS3*^+^CD4^+^ T cells in biliary tumors by scRNAseq (Figures S7A and S7B). Using *in situ* hybridization, we corroborated these results finding not only the presence of CD3^+^CD4^+^SOCS3^+^ T cells in the human BTC tissue sections, but also their co-localization with *HAVCR2*^+^*SPP1*^+^*CD68*^+^ cells in the tumor microenvironment, demonstrating that *HAVCR2*^+^*SPP1*^+^*CD68*^+^ macrophages are located closer to CD3^+^CD4^+^*SOCS3*^+^ T cells than non-*SPP1/HAVCR2*^*+*^ *CD68*^+^ macrophages (p = 0.04) (Figures 5J and S7C–S7F).

### DISCUSSION

Circulating and tissue-resident myeloid cells are known to be heterogeneous in cancer patients, having immune-modulating functions ranging from being tumor promoting to tumor suppressing (DeNardo et al., 2010; Gabrilovich et al., 2012; Hegde et al., 2021). An understanding of immunosuppressive capacity of monocytes, MDSC, M2 macrophages, and TAMs is emerging, along with the heterogeneity of myeloid phenotypes within different tumor types (Cheng et al., 2021; Gallina et al., 2006; Mantovani et al., 2017; Ostrand-Rosenberg and Fenselau, 2018; Trovato et al., 2019). By using multiplexed single-cell transcript and protein profiling of PBMCs, we identified circulating monocytes as a hallmark of cancer and of insensitivity to immunotherapy. While these monocytes share some features of MDSC and M2 macrophages, they do not conform to these classifiers and lack expression of MDSC/M2-associated genes such as *ARG1*, *VEGFA*, and *IDO1*. The monocyte subpopulation associated with anti-PD-1 insensitivity (CD14_CTX_) has increased expression of chemokines and molecules involved in extracellular matrix digestion, which could facilitate migration into the tumor microenvironment and could represent a precursor of TAMs. This result was further supported by overall highly correlated gene signatures, with downregulation of genes related to extravasation, in TAMs from primary cholangiocarcinoma tumors. Incongruous findings have been observed regarding the association of TAMs with biliary cancer patient prognosis, highlighting the challenge in applying one label to a heterogenous group of cells that can have anti- or pro-oncogenic phenotypes (Loeuillard et al., 2019). Our observation that alteration in monocytes was associated with clinical response to anti-PD-1 aligns with findings in melanoma patients, although we observed clinical associations with circulating monocyte populations emerging on treatment rather than being present at baseline (Krieg et al., 2018). While we observed that dendritic cells increased in frequency in non-responders following anti-PD-1, this may be secondary to the altered frequency of monocyte sub-populations.

Using our single-cell multi-omic data, we developed cell surface markers and gene signatures of CD14_CTX_ that can be used to assess these cells by more conventional means and could be further explored as a circulating biomarker or a target for future therapies. First, our CITE-seq data nominates Tim3 and CD29 as more specific combinatorial markers to identify these circulating myeloid cells within BTC patients. Furthermore, CD14_CTX_ express certain molecules associated with immunosuppression such as *CXCL8*, *TGFB1*, and *IL6*, which could be targeted by anti-cancer therapy independently. The CD14_CTX_ gene signature correlated with poor prognosis in other CPI-insensitive cancers such as prostate and colorectal cancer and in patients treated with CPI. In addition, CD14_CTX_ aligned with *SPP1* (osteopontin)-expressing TAMs, a broadly expressed, pleiotropic molecule, involved in chemotaxis, anti-apoptosis, and maladaptive wound-healing response, with both pro- and anti-inflammatory roles (Denhardtet al., 2001; Muliaditan et al., 2018). SPP1 expression correlates with poor prognosis in many cancer types, including biliary cancer, and SPP1^+^ TAMs have been identified in other CPI-insensitive diseases including colorectal cancer (Muliaditan et al., 2018; Sulpice et al., 2013; Zhang et al., 2020a; Zheng et al., 2018). Finally, we demonstrated that CD14_CTX_ could induce SOCS3 expression in CD4^+^ T cells, a known negative regulator of cytokine signaling and mediator of T cell ‘‘immune paralysis’’ (Croker et al., 2003; Jiang et al., 2017; Sckisel et al., 2015). T cell unresponsiveness induced in T cells by cancer-associated myeloid cells is an emerging mechanism of immunosuppression distinct from those mediated by other immune checkpoint pathways (Emmons et al., 2021). Here, in the context of biliary cancer, we demonstrated that circulating SOCS3^+^CD4^+^ T cells also exhibited immune paralysis following stimulation *in vitro*. Targeting these immunosuppressive myeloid populations driving T cell paralysis, in combination with CPI, presents a future avenue for overcoming CPI insensitivity and improving outcomes in patients with BTC.

### Limitations of the study

While our study provides important insights into the circulating myeloid cells of BTC patients and mechanisms of CPI response and resistance in BTC, the single-cell dataset is derived from a small cohort. Therefore, we sought to validate our observations with independent cohorts of cholangiocarcinoma tumors through assessing additional patients as well as examining a cohort from the TCGA dataset. The overall low response rate to CPI in BTC likely reflects multiple mechanisms of resistance, which could be further elucidated by examining additional patient cohorts. While we found that induction of CD14_CTX_ corresponds with resistance to CPI, the mechanisms controlling the induction of responder versus non-responder myeloid sub-populations remain to be determined. Lastly, we demonstrated that the plasma from patients with BTC can induce SOCS3 in CD4^+^ T cells and that myeloid cells from BTC patients can suppress T cell proliferation and cytokine production, but the factor(s) inhibiting T cells also remain to be identified.

## STAR★METHODS

### RESOURCE AVAILABILITY

#### Lead contact

Further information and requests for resources and reagents should be directed to and will be fulfilled by the lead contact, Lawrence Fong, lawrence.fong@ucsf.edu.

#### Materials availability

This study did not generate new unique reagents or biological materials.

#### Data and code availability

- Single-cell RNA-seq, CITEseq, and bulk RNA-seq data have been deposited at GEO: GSE210067 and are publicly available as of the date of publication. Accession numbers are listed in the key resources table.
- All original code has been deposited in github and is publicly available as of the date of publication. Access information is listed in the key resources table. The software used in this study is described in the following sections and the key resources table in detail.
- Any additional information required to reanalyze the data reported in this paper is available from the lead contact upon request.

### EXPERIMENTAL MODEL AND SUBJECT DETAILS

Peripheral blood mononuclear cells were obtained from patients pre- and on-treatment (per UCSF institutional review board (IRB) #15–18420) from the clinical trial of staggered or simultaneous GM-CSF and anti-PD-1 (pembrolizumab) (n = 9 total, 4 women and 5 men, age range 53–73 years). Patients, per eligibility criteria, had advanced biliary tract cancer previously treated with chemotherapy, and no active uncontrolled infections. BTC patients started treatment with anti-PD-1 (administered intravenously starting on cycle 1 day 1 (C1D1) and repeating every 3 weeks) and subsequently received GM-CSF (administered subcutaneously in cycles 2 and 3 for 14 days each) (Kelley RK et al., 2018). We profiled blood samples from BTC patients from baseline, 1 week following anti-PD-1, and 3 weeks following anti-PD-1 immediately prior to cycle 2; this report does not examine effects of GM-CSF, as patients received GM-CSF after the collection of these sample timepoints. For the purposes of this study, responders (n = 4) were characterized as patients that had an objective partial response or stable disease by imaging, resulting in progression-free survival for 6 months or longer. Non-responders (n = 5) were patients that did not have objective tumor responses and/or who had progression-free survival less than 6 months. Tumor samples were collected from patients biopsied as part of the Phase II clinical trial and from patients undergoing standard-of-care resections and consented under the UCSF Hepatobiliary Tissue Bank and Registry (IRB #12–09576) (n = 4 total, 3 men and 1 woman, age range 37–60 years) Healthy donor PBMCs were collected from age and gender-matched healthy donors as part of the Cancer Immunotherapy Biobanking protocol and the Immune Cell Census (IRB #15–16385 and #19–27147, respectively; n = 8 total, 4 women and 4 men, age range 46–77 years); healthy donor samples reflect one timepoint, with multiple independent replicates sequenced. Informed consent was obtained from all patients for participation in the listed trials and for use of blood and tumor samples in research studies. Patients’ and healthy donors’ age, sex, gender, race, ethnicity, and additional tumor-related characteristics are provided in Tables S1 and S7.

### METHOD DETAILS

#### Processing of samples, scRNAseq, and CITE-seq

Blood samples were processed using ficoll (Cytiva); after centrifugation, the peripheral blood mononuclear cell (PBMC) layer was isolated and cryopreserved in cell media with human serum and DMSO. Previously frozen PBMCs from healthy donor and BTC patients were thawed using media containing RPMI, heat-inactivated sterile filtered human serum, penicillin-streptomycin, non-essential amino acids, sodium pyruvate, and L-glutamine (CHM media). Samples were then incubated for DNAse I (15 units/mL, Roche) before washing and counting. 1 × 10^6^ cells from 16 unique samples were combined and stained with one pooled cocktail containing 99 AbSeq antibody-oligonucleotide conjugates (Table S2, BD Biosciences) per standard protocols (Olvera et al., 2018), following preincubation with TruStain FcX (Fc Receptor Blocking Solution, Biolegend). Samples from different individuals and timepoints were randomly mixed across experiments to minimize batch and confounding effects (Figures S1A and S1B). Droplet-based single cell RNA sequencing (scRNAseq) was performed using the 10× Genomics Chromium Single Cell 3ʹ Reagent Kits v3, according to manufacturer instructions. For tumor tissues, samples were digested in RPMI containing Collagenase I & II (0.1 mg/mL, Thermo Fisher Scientific) and DNAse I, minced, and digested for one hour using the GentleMACS system (Miltenyi Biotec). Isolation of live cells was performed using MACS LS columns (Miltenyi Biotec). scRNAseq of tumor samples was completed on fresh material with 10× 5′ version 1 kits. All sequencing was performed on an Illumina NovaSeq S4 sequencer with paired end 200 base pair read length and 25,000 reads per droplet.

#### RNA extraction and bulk RNA sequencing

RNA extraction and bulk RNA sequencing were performed to obtain single nucleotide polymorphism information for sample deconvolution. The RNeasy Mini Kit (Qiagen) was used to extract RNA from minimum 2.5 × 10^5^ cells per PBMC sample. cDNA was prepared using methods previously described, with the Smart-seq2 protocol (Picelli et al., 2014), and libraries were prepared using Nextera XT DNA Sample Preparation Kit. Bulk RNA from each sample was sequenced at a depth of at least 2 × 10^7^ reads per cell on the Illumina Novaseq S4 and aligned to human genome build 38 with STAR (Dobin et al., 2013). Pre-processing of aligned sequencing data and identification of single nucleotide polymorphisms was performed using the Genome Analysis Toolkit (McKenna et al., 2010) as previously described (Bunis et al., 2021). We used demuxlet (Kang et al., 2018) (https://github.com/statgen/demuxlet) for sample deconvolution of multiplexed PBMC samples, removing any samples that lacked high confidence in sample identification.

#### Pre-processing of scRNAseq data

CellRanger version 3.1.0 (10× Genomics, Genome Build: GRCh38 3.0.0) was used to align the raw sequencing data. The ADT library sequences were aligned to a customized reference genome provided by BD containing the oligonucleotide sequences corresponding to each antibody. We used the SCANPY (Wolf et al., 2018) data analysis pipeline for pre-processing and analysis of scRNAseq data, with the following software versions: scanpy 1.4.6, anndata 0.7.1, umap 0.4.1, numpy 1.18.1, scipy 1.4.1, pandas 1.0.3, scikitlearn 0.21.2, statsmodels 0.10.1, python-igraph 0.8.0, and louvain 0.6.1. We applied the following cutoffs for filtering high quality cells: <20% mitochondrial genes, >100 and <2500 genes expressed per cell, and excluded platelets, red blood cells, and doublets. We filtered out ribosomal genes and genes detected in less than three cells. Following sequencing alignment, pre-processing, quality control, and doublet removal, we recovered over 230,000 cells from all samples combined, corresponding to greater than 5,000 cells per sample. We log_2_ plus one transformed, normalized the data to 10,000 counts per cell, regressed out gender, percent mitochondrial genes, and number of gene counts, and scaled genes to unit variance. We performed batch correction using ComBat (Johnson et al., 2007) and highly variable genes present in greater than 4 of 11 independent experiments, using the SCANPY function for highly variable genes, and ran principle component analysis with SCANPY. We then performed k-nearest neighbor graph construction and clustering on gene expression data; for analysis of all immune cells, we clustered cells with a resolution of 1.0. We re-clustered on myeloid or T cells individually, removing any contaminating cells (non-myeloid or non-T cell), for myeloid, we used a resolution of 0.3; for T cells, we used resolution 0.6. For protein data, we processed the data by log_2_ plus one transformation, regressing out batch, and scaled as for RNA. For the fresh tumor tissue dataset, we applied the same pre-processing pipeline and used previously established gene lists used for the annotation of cells in cholangiocarcinoma including immune and non-immune cells (Zhang et al., 2020b) and identified four myeloid clusters, three lymphocyte clusters, and three malignant cell clusters. We independently re-clustered on the intra-tumoral myeloid cells and T cells using a resolution of 0.3 and 1, respectively.

#### scRNAseq analysis

We used the SCANPY embedded function to determine top differentially expressed genes for all immune cells, T cells and myeloid sub-types; for further analysis, we used MAST (see Statistical Analysis). Cell types were annotated using commonly expressed protein (Figure S1C and Table S2) and transcript markers (Figure S1D). CITE-seq generally produced strong correlation (R = 0.40 to 0.71, p = 1.25 × 10^−7^ to 9.31 × 10^−3^, inclusive of all values except for CD4) between protein and RNA expression for canonical immune cell type markers across individual samples, except in genes that have low levels of transcript abundance such as *CD4* (Figures S1E–S1G). We used COMET (Delaney et al., 2019) to identify combinatorial gene expression by analyzing a subset of 1000 equally sampled cells from the CD14_CTX_, CD14_APC,_ and CD14_IFL_ populations and running three iterations with different random samples. We then used this list to identify highly ranked gene pairs that were cell surface proteins contained in the CITE-seq panel. Trajectory analysis was performed using Monocle v2.10.1 (Qiu et al., 2017; Trapnell et al., 2014), using a sub-sample of maximum 10,000 total cells with equal cell number sampled from each cell type. For gene signature comparisons between circulating immune cells and intra-tumoral immune cells, we created a matrix of pseudobulk expression for each cell type and then performed correlation analysis on pseudobulk gene expression profiles. For cross-data set comparison and clustering of intra-tumoral and circulating myeloid cells, we used Harmony to process data (Korsunsky et al., 2019) and partition-based graph abstraction (PAGA) (https://github.com/theislab/paga) to demonstrate the connectivity of clusters (Wolf et al., 2019). For gene ontology analysis, we used an immune-specific pathway database (Li et al., 2014), and used an adjusted p value cutoff of <0.05 and a log fold-change of 0.5 (as determined by MAST, as described under Statistical Analysis) for the genes from each monocyte sub-population. For over-representation analysis, we used the enricher function from clusterprofiler (http://www.bioconductor.org/packages/release/bioc/html/clusterProfiler.html) (Yu et al., 2012) and cnetplot function from DOSE (https://bioconductor.org/packages/release/bioc/html/DOSE.html) (Yu et al., 2015).

#### Flow cytometry and *in vitro* experiments

PBMC samples were thawed as described for scRNAseq, incubated with TruStain FcX (Biolegend), and stained with LIVE/DEAD Fixable Near-IR Dead Cell Stain (Invitrogen), followed by surface antibody staining. For CD68, SOCS3, and cytokine staining, we performed intracellular staining using the Intracellular Fixation & Permeabilization kit (eBioscience). Data was acquired using the LSRFortessa cytometer (BD Biosciences). We performed FACS with the gating schema described in the Results section and Figures S6A and S6B to obtain the sorted populations from healthy donor and patient PBMCs, using a FACSAria Fusion (BD Biosciences). In T cell/myeloid cell co-cultures, cells were plated at 1:1 ratio for effector T cells:myeloid population, with 2 × 10^5^ T total cells per well, in CHM media and 10 units IL-2. Cells were harvested on day 6 for analysis with flow cytometry. Intracellular SOCS3 staining was performed using an unconjugated primary (Cell Signaling) and a fluorescently conjugated secondary antibody (Jackson ImmunoResearch). For T cell stimulation experiments, we used anti-CD3/CD28 beads (ThermoFisher Scientific) in culture for 3 days before harvest; protein transport inhibitor cocktail (eBioscience) was added to co-cultures for 4 h before harvest and intracellular cytokine staining. Complete information for antibodies used is available in Key Resources Table. For suppression assays, sorted T cells were stained with CFSE (CellTrace, Invitrogen) per manufacturer instructions prior to co-culture with monocytes.

#### Tissue staining and image analysis

RNAscope (Advanced Cell Diagnostics, ACD) *in situ* hybridization and immunofluorescence were performed on 4μm FFPE sections obtained from control tonsil and from biopsies collected from BTC patients treated on the clinical trial. Tissues were pre-treated with target retrieval reagents and protease to improve target recovery based on guidelines provided in the RNAscope Multiplex Fluorescent Reagents Kit v2 Assay protocol. mRNA expression was demonstrated using probes for *CD68*, *SOCS3*, *SPP1*, and *HAVCR2* (ACD). Probes were hybridized with Opal 7-Color Manual IHC Kit (PerkinElmer) to produce discrete points of light. Samples were then stained for CD4 (Thermo Fisher Scientific) and CD3 (Abcam) and with the AF488 and AF555-conjugated secondary antibodies given in Key Resources Table. Tissues were counterstained with DAPI. Slides were imaged using TCS SP8 X white light laser inverted confocal microscope (Leica Microsystems, Inc). The ARK Python library (Greenwald et al., 2022) was used to perform cell segmentation based on the DAPI (nuclear) stain, extract single-cell marker counts, and generate a CSV file of cell size-normalized and arcsinh-transformed single-cell marker counts. A re-scale factor of 0.5 was applied to format 40× image data for use with a segmentation model trained on 20× data. Microsoft Excel was used to produce histograms and biaxial scatterplots that were then used to gate image-specific CSV files of cell populations of interest. These cell population CSVs were imported into CytoMAP (Stoltzfus et al., 2020), which we used to plot cell centroids colored by cell type. CytoMAP’s Calculate Distance tool was used to calculate average distances between cells. Median distances were calculated for each image and compared with a one-sided paired t-test.

### QUANTIFICATION AND STATISTICAL ANALYSIS

#### Statistical analysis

For differential expression analysis, we used the embedded SCANPY function to identify differentially expressed genes in each cluster compared to the union of the rest of the clusters which uses Benjamini-Hochberg (Hochberg and Benjamini, 1990) to control the false discovery rate. For specific comparisons of differential gene expression between cell types, we used MAST to calculate fold change and significance, based on a model incorporating cellular detection rate (based on number of genes per cell), gender, and patient as covariates (Finak et al., 2015). For frequency proportions, weighted least squares was used to adjust for number of cells sequenced in each individual and Benjamini-Hochberg method was used to adjust p-values for multiple comparisons. To assess the correlations of the frequency of cell types, we used Spearman’s rank correlation coefficient. Flow cytometry data was analyzed with FlowJo (FlowJo Software for Mac Version 10, 2019) for data analysis. A two-sample t-test was used to compare frequency of cell types between patients and healthy donors, and for analyses with multiple groups, one-way ANOVA was performed, using GraphPad Prism version 8.3.0. Additional details such as statistical test used, number of samples, and p-values can be located in figure legends and in Tables S3 and S4, S5, and S6.

#### Survival analysis

Raw gene expression counts were downloaded from cholangiocarcinoma (Farshidfar et al., 2017), prostate cancer (Cancer Genome Atlas Research, 2015), and colon cancer (Cancer Genome Atlas, 2012) datasets using The Cancer Genomics Cloud (Lau et al., 2017); additional clinical metadata was downloaded from cBioportal (Cerami et al., 2012). Overall survival (OS) and disease-free survival (DFS) were defined as from the time of collection of tissues to the date of death or last follow-up and estimated by the Kaplan-Meier method. For the checkpoint inhibitor-treated datasets, we downloaded data from the phase 3 JAVELIN Renal 101 trial (n = 886; NCT02684006; (Choueiri et al., 2020; Motzer et al., 2020)) and from baseline biopsies prior to pembrolizumab (anti-PD-1) treatment in melanoma patients (n = 112; Validation Cohort 2 (Jerby-Arnon et al., 2018)) and for both datasets, used progression-free survival as the clinical endpoint. We started with the top 20 differentially expressed genes in CD14_CTX_, as determined by MAST, and then used only genes found in both datasets. We use a normalized z score for each gene, which is calculated by (raw gene expression – mean expression)/standard deviation of expression; then the composite score was calculated as the linear combination of the coefficients estimated based on the multivariable Cox proportional hazards (CPH) model (which includes all the top 20 genes) multiplied by the corresponding gene expression values (Yasrebi et al., 2009). When fitting the CPH model, panelized regression with LASSO (least absolute shrinkage and selection operator) method was applied to avoid overfitting (Waldron et al., 2011). We compared the OS between patients who had the higher composite score (above the median) versus those with the lower score by log rank test.

### ADDITIONAL RESOURCES

Peripheral blood and tissue samples obtained from BTC patients on a phase II clinical trial with the ClinicalTrials.gov identifier NCT02703714.

## Supplementary Material

1

2

3

4

## Figures and Tables

**Table:** KEY RESOURCES TABLE

REAGENT or RESOURCE	SOURCE	IDENTIFIER

Antibodies		

SOCS3	Cell Signaling Technology	Cat# 52113; RRID: AB_2799408
BV510-conjugated CD14	Biolegend	Cat# 367123; RRID: AB_2716228
BV421-conjugated anti-rabbit secondary	Jackson ImmunoResearch Laboratories, Inc.	Cat# 111–675-144; RRID: AB_2651074
BV650-conjugated CDS	Biolegend	Cat# 344729; RRID: AB_2564509
BV785-conjugated CD3	Biolegend	Cat# 300472; RRID: AB_2687178
PE-Cy7-conjugated Tim3	Biolegend	Cat# 345014; RRID: AB_2561720
BUV395-conjugated CD45	BD Biosciences	Cat# 563791; RRID: AB_2744400
AF647-conjugated CD29	Biolegend	Cat# 303018; RRID: AB_2130080
APC-R700-conjugated CD56	BD Biosciences	Cat# 565139; RRID: AB_2744429
PE-conjugated CD4	Biolegend	Cat# 300508; RRID: AB_314076
AF4SS-conjugated CD4	Biolegend	Cat# 317420; RRID: AB_571939
BV711-conjugated IL-2	BD Biosciences	Cat# 563946; RRID: AB_2738501
PE-conjugated TNFα	Biolegend	Cat# 502909; RRID: AB_315261
AF647-conjugated IFNγ	Biolegend	Cat# 502516; RRID: AB_493031
BV421-conjugated mouse IgG1, κ isotype control	Biolegend	Cat# 400158; RRID: AB_11150232
AF647-conjugated mouse IgG1, κ isotype control	Biolegend	Cat# 400155; RRID:AB_2832978
PE-conjugated mouse IgG1, κ isotype control	Biolegend	Cat# 400112; RRID: AB_2847829
BV711-conjugated mouse IgG1, κ isotype control	Biolegend	Cat# 400167; RRID: AB_11218607
CD4	Thermo Fisher Scientific	Cat# MA5–12259; RRID: AB_10989399
AF488 anti-mouse secondary	Abcam	Cat# ab150105; RRID: AB_2732856
CD3	Abcam	Cat# ab16669; RRID: AB_443425
AF555 anti-rabbit secondary	Southern Biotech	Cat# 4050-32; RRID: AB_2795963

Biological samples		

Biliary tract cancer patient blood samples	This study	NCT02703714
Biliary tract cancer tumor samples	This study	This study
Healthy donor blood samples	This study	This study

Chemicals, peptides, and recombinant proteins		

Ficoll	Cytiva	Cat #17144003
Human serum	Sigma Aldrich	Cat #H6914
DMSO	Sigma Aldrich	Cat #D2650
DNAse I	Roche	Cat #4536282001
Collagenase I	Thermo Fisher Scientific	Cat #17018029
Collagenase II	Thermo Fisher Scientific	Cat #17101015
LIVE/DEAD Fixable Near-IR Dead Cell Stain	Invitrogen	Cat #L10119
TruStain FcX Fc Receptor Blocking Solution	Biolegend	Cat #422302
Intracellular Fixation & Permeabilization kit	eBioscience	Cat #00–5523-00
IL-2	Peprotech	Cat #200-02
Dynabead Human T-Activator CD3/CD28 beads	Thermo Fisher Scientific	Cat #11131D
Protein transport inhibitor cocktail	eBioscience	Cat #00–4980-93
CFSE	CellTrace, Invitrogen	Cat #C34554
SOCS3 RNA probe	ACDBio	Cat #469931
SPP1 RNA probe	ACDBio	Cat #420101-C2
REAGENT or RESOURCE	SOURCE	IDENTIFIER
HAVCR2 RNA probe	ACDBio	Cat #560681-C3
CD68 RNA probe	ACDBio	Cat #560591-C4

Critical commercial assays		

AbSeq antibody-oligonucleotide conjugates	BD Biosciences	Custom panel, see Table S2 for antibody and clone information
Chromium Single Cell 3’ Reagent Kits v3	10X Genomics	Cat #1000075
Chromium Single Cell 5’ Reagent Kits v1	10X Genomics	Cat #1000006
MACS LS columns	Miltenyi Biotec	Cat #130–042-401
RNeasy Mini Kit	Qiagen	Cat #74106
Nextera XT DNA Sample Preparation Kit	Illumina	Cat #FC-131–1096
RNAscope Multiplex Fluorescent Reagents Kit v2 Assay	Advanced Cell Diagnostics	Cat #ACD 323110
Opal 7-Color Manual IHC Kit	PerkinElmer	Cat #NEL811001KT
RNAscope Pretreatment reagents	Advanced Cell Diagnostics	Cat #ACD 322380
RNAscope Wash Buffer Reagents	Advanced Cell Diagnostics	Cat #ACD 310091

Deposited data		

scRNAseq, CITEseq, and bulkRNAseq data	This study	GEO: GSE210067
TCGA datasets (prostate cancer, cholangiocarcinoma, colorectal cancer)	Farshidfar et al. (2017); Cancer Genome Atlas Research (2015); Cancer Genome Atlas (2012); Lau et al. (2017)	The Cancer Genomics Cloud, cBioportal
JAVELIN Renal 101 cohort	Choueiri et al. (2020); Motzer et al. (2020)	NCT02684006
Melanoma “validation cohort 2’’	Jerby-Arnon et al. (2018)	N/A

Software and algorithms		

STAR (v2.7.3a)	Dobin et al. (2013)	https://github.com/alexdobin/STAR
Genome Analysis Toolkit (v4.1.5.0)	McKenna et al. (2010)	https://gatk.broadinstitute.org/hc/en-us
Demuxlet	Kang et al. (2018)	https://github.com/statgen/demuxlet, https://github.com/hyunminkang/cramore
CellRanger (v3.1.0, Genome Build: GRCh38 3.0.0)	10x Genomics	https://support.10xgenomics.com/single-cell-gene-expression/software/overview/welcome
Scanpy (v1.4.6)	Wolf et al. (2018)	https://github.com/scverse/scanpy
MAST (1.12.0)	Finaketal. (2015)	https://github.com/RGLab/MAST
COMET (web interface, no version)	Delaney et al. (2019)	http://www.cometsc.com/
Monocle (v2.10.1)	Qiu et al. (2017); Trapnell et al. (2014)	http://cole-trapnell-lab.github.io/monocle-release/docs/
Harmony (v0.0.4)	Korsunsky et al. (2019)	https://github.com/slowkow/harmonypy
PAGA (Scanpy v1.4.6)	Wolf et al. (2019)	https://github.com/theislab/paga
Clusterprofiler (v3.18.1)	Yu et al. (2012)	http://www.bioconductor.org/packages/release/bioc/html/clusterProfiler.html
DOSE (v3.16.0)	Yu et al. (2015)	https://bioconductor.org/packages/release/bioc/html/DOSE.html
ARK (v0.2.11)	Greenwald et al. (2022)	https://github.com/angelolab/ark-analysis
Survival analysis code	This study	https://github.com/mlizhangx/TCGA_SurvivalAnalysis
CytoMAP (v1.4.21)	Stoltzfus et al. (2020)	https://gitlab.com/gernerlab/cytomap
FlowJo (v10)	BD Life Sciences	https://www.flowjo.com/
Prism (v8.3.0)	GraphPad	https://www.graphpad.com/
Leica Application Suite X (v3.3.3.16958)	Leica Microsystems, Inc	https://www.leica-microsystems.com/products/microscope-software/p/leica-las-x-ls/

Other		

GentleMACS	Miltenyi Biotec	Cat #130–096-427
Illumina NovaSeq S4 sequencer	Illumina, USA	Not available
LSRFortessa cytometer	BD Biosciences	Not available
FACSAria Fusion	BD Biosciences	Not available
RNAscope	Advanced Cell Diagnostics	Not available
TCS SP8 X microscope	Leica Microsystems, Inc	Not available

## Highlights

- Multi-omic single-cell sequencing reveals circulating myeloid states in biliary cancer
- The CD14_CTX_ monocyte state is associated with resistance to checkpoint inhibitors (CPI)
- The CD14_CTX_ molecular signature associates with poor prognosis across cancer types
- CD14_CTX_ induce CD4^+^ T cell immune paralysis, a potential mechanism of CPI resistance
