# Supplementary material for: Circulating monocytes associated with anti-PD-1 resistance in human biliary cancer induce T cell paralysis
Source: Cell Rep. Author manuscript; Available in PMC 2023 Mar 29. (PMC10060099; doi:10.1016/j.celrep.2022.111384)
Supplement: 1 [file NIHMS1837779-supplement-1.pdf]

Cell Reports, Volume *40*

Supplemental information

Circulating monocytes associated with anti-PD-1 resistance in human biliary cancer induce T cell paralysis

Bridget P. Keenan, Elizabeth E. McCarthy, Arielle Ilano, Hai Yang, Li Zhang, Kathryn Allaire, Zenghua Fan, Tony Li, David S. Lee, Yang Sun, Alexander Cheung, Diamond Luong, Hewitt Chang, Brandon Chen, Jaqueline Marquez, Brenna Sheldon, Robin K. Kelley, Chun Jimmie Ye, and Lawrence Fong

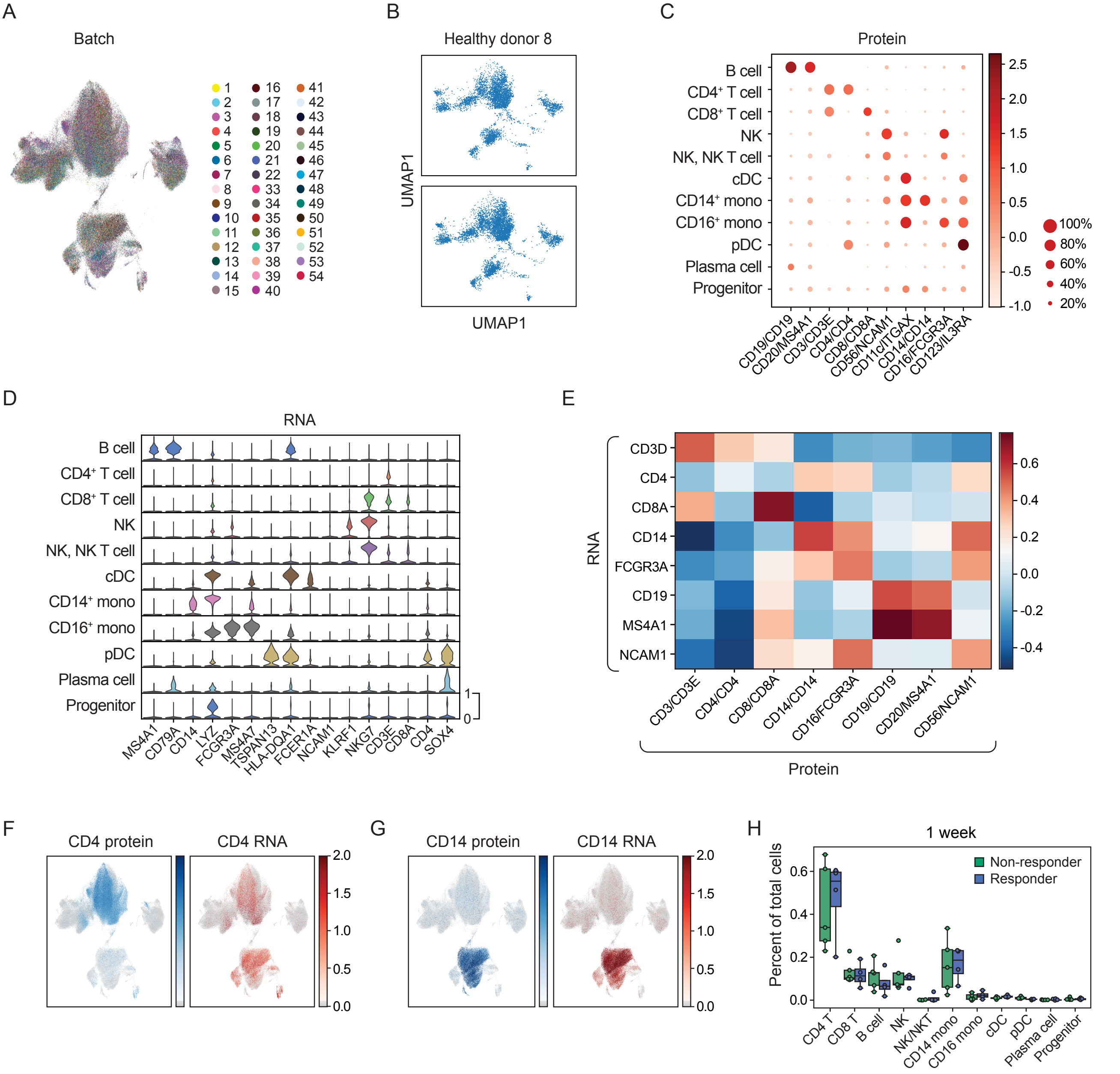

Figure S1. Characterization of circulating immune cells with CITE-seq, related to Figure 1. (A) UMAP colored by experimental batch (10x well replicate). (B) Individual replicates are shown for one example (Healthy Donor-8) to demonstrate reproducibility across batches. (C) Expression of proteins used to classify immune cell clusters are shown by percentage of cells with expression above the zero threshold (dot size) and mean expression (color). (D) For each immune cell class, expression of each gene is shown using a standard scale (for each gene, minimum is subtracted and then divided by its maximum). (E) Correlation plot of transcript (y-axis) and corresponding protein expression (x-axis) by pseudobulk expression data for all immune cells in the dataset. Legend shows value for the Spearman correlation coefficient. (F-G) Protein (left panels) and transcript (right panels) expression overlaid on UMAP plots, for CD4 (F) and CD14 (G). (H) Percent of each cell type out of total immune cells in responders (n=4) and non-responders (n=5) one week following anti-PD-1 administration. Boxes denote inter-quartile range (IQR) while bars denote 25% - 1.5xIQR and 75% + 1.5xIQR.

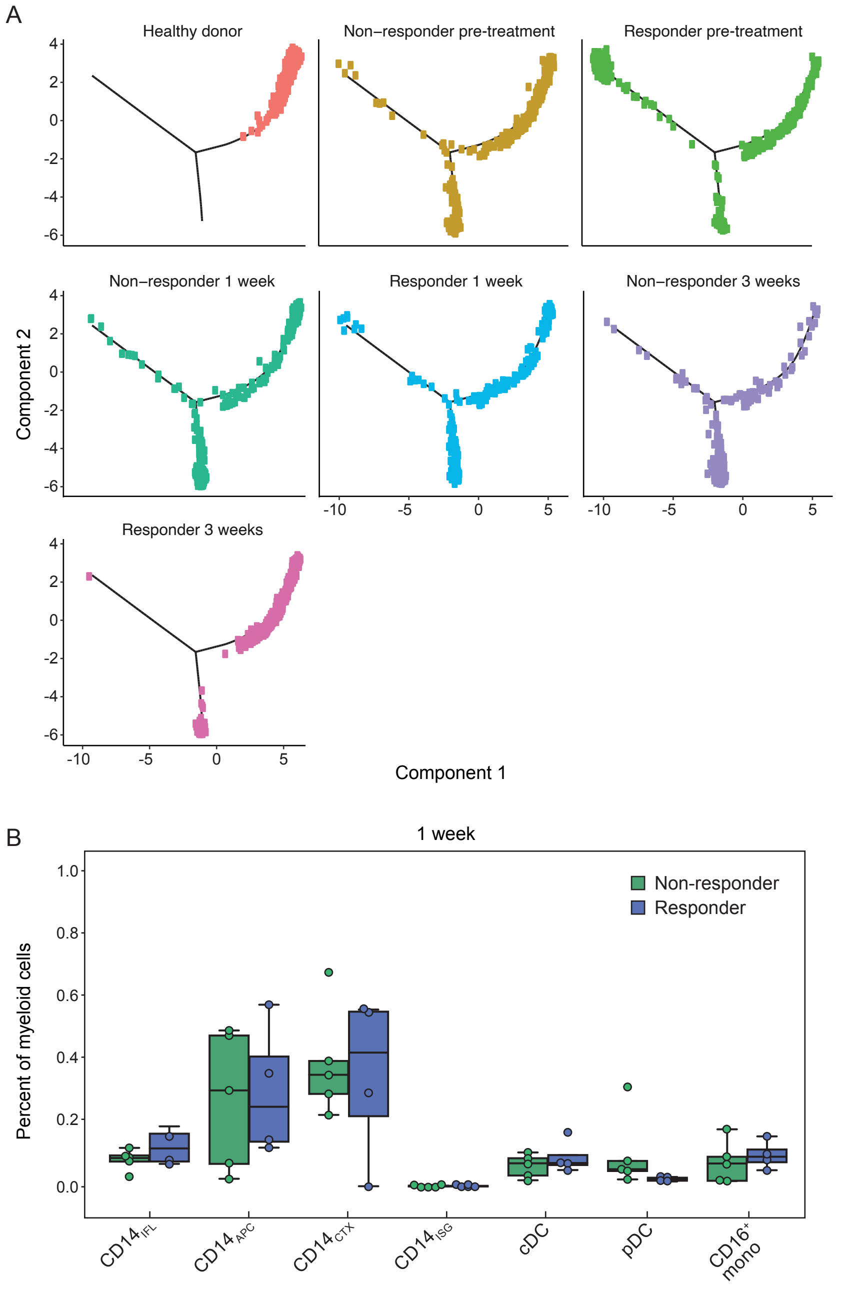

Figure S2. Monocytes sub-populations are differentially distributed in latent time and dynamic over time with treatment, related to Figure 3. (A) Monocyte sub-populations are shown ordered in latent time for each timepoint/response category. (B) Percent of each cell type out of all myeloid cells in responders (n=4) and non-responders (n=5) one week following anti-PD-1 administration. Boxes denote inter-quartile range (IQR) while bars denote 25% - 1.5xIQR and 75% + 1.5xIQR.

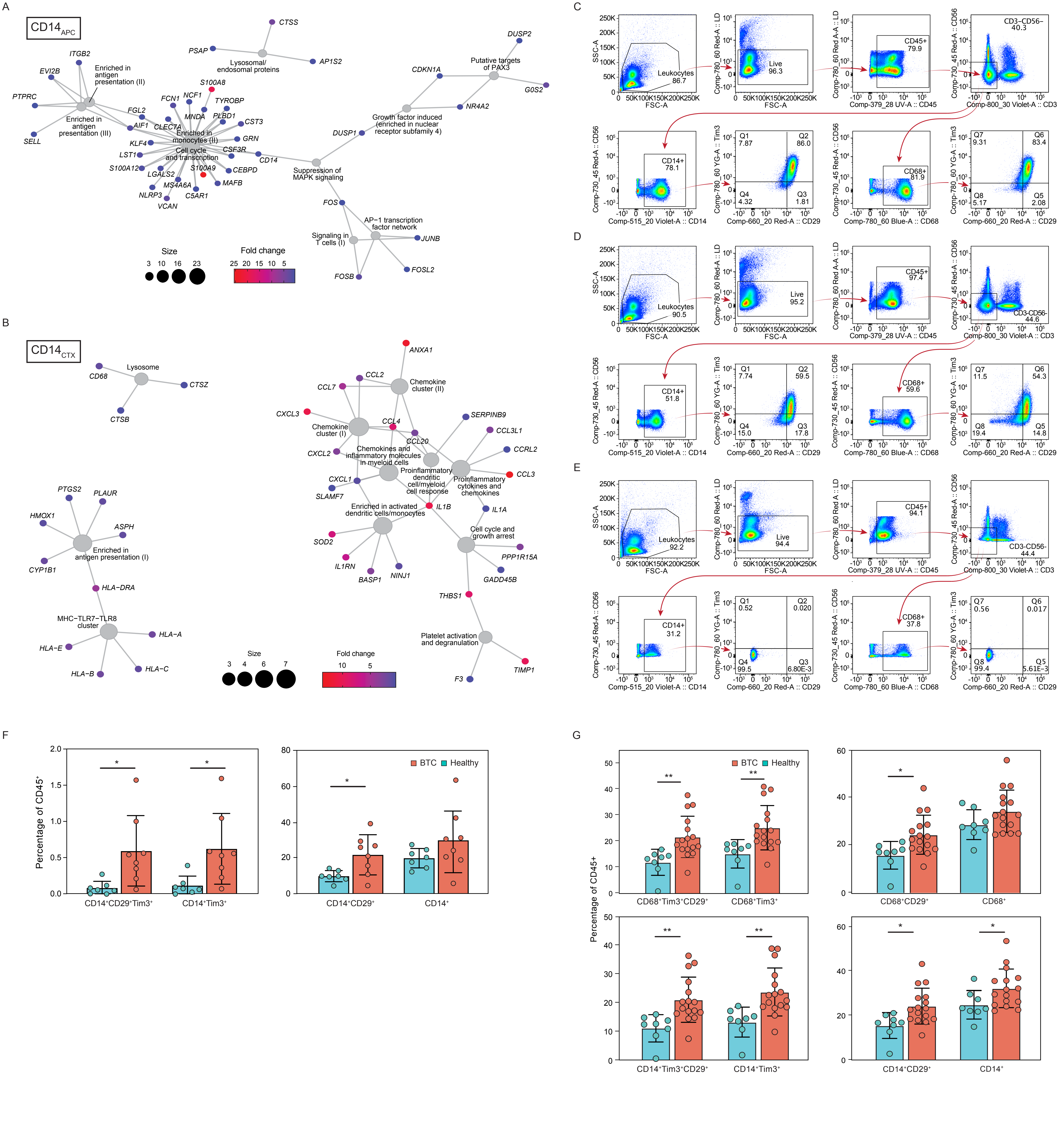

Figure S3. CPI responders and non-responders have diverging myeloid sub-populations, related to Figure 4. (A-B) Immune pathways are plotted as a network with genes assigned to each pathway and differentially expressed in $CD14_{APC}$ (A) or $CD14_{CTX}$ (B). Number of genes in each pathway is shown by the size of the pathway circle and fold change is shown by the heatmap in the legend. (C-E) Gating strategy for flow cytometry experiments staining for $CD68^+$ and $CD14^+$ $CD29^+Tim3^+$ cells, shown for representative BTC patient peripheral blood sample (C), healthy donor sample (D), and isotype control for CD29 and Tim3 staining (E). (F) Bar plots of each myeloid population gated on CD14 and calculated as percentage of total $CD45^+$ circulating immune cells as analyzed by flow cytometry of peripheral blood cells from BTC patients (n=8) and healthy donors (n=7). * = p<0.05, error bars denote standard deviation. (G) Bar plots of each myeloid population gated on CD68 (top panels) or CD14 (bottom panels) and calculated as percentage of total $CD45^+$ circulating immune cells as analyzed by flow cytometry of peripheral blood cells from BTC patients (n=16) and healthy donors (n=8). * = p<0.05, ** = p< 0.01, error bars denote standard deviation.

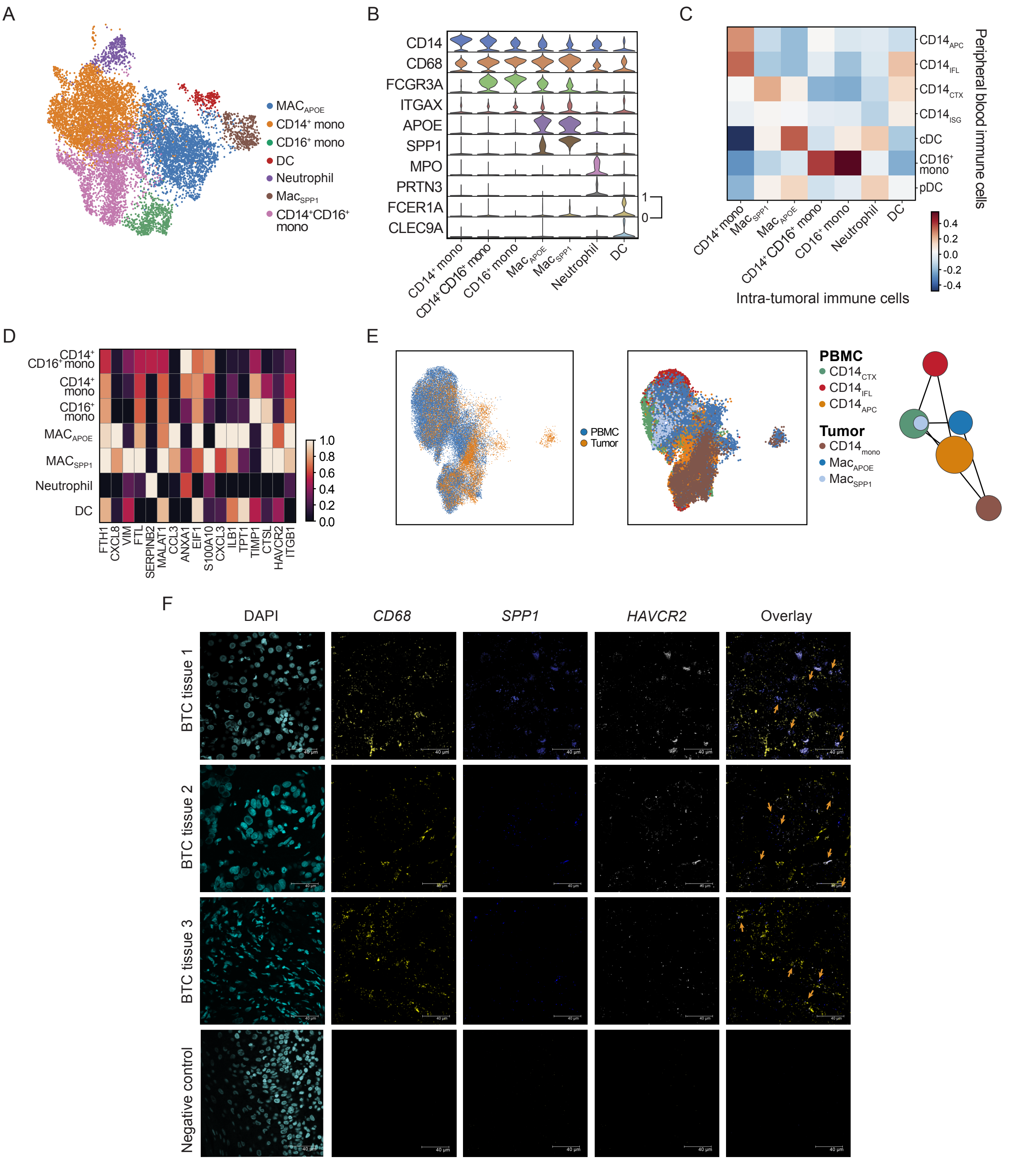

Figure S4. Intra-tumoral BTC myeloid populations correlate with circulating CD14$_{CTX}$, related to Figure 4. (A) UMAP colored by myeloid cell sub-types present in biliary cancer tumor dataset. (B) Expression of genes for phenotypic markers are shown for myeloid cell sub-types in the tumor dataset using a standard scale (for each gene, minimum is subtracted and then divided by its maximum). (C) Heatmap of Pearson correlation coefficient values for gene signatures using pseudobulk gene expression data for each myeloid cell sub-type from peripheral blood and intra-tumoral datasets. Legend shows R value for each correlation. (D) Mean expression of CD14$_{CTX}$ hallmark genes in myeloid cells isolated from biliary tumors. (E) UMAP plots of combined intra-tumoral and circulating monocytes and macrophages from biliary tract cancer patients, followed batch correction, colored by compartment (left plot) or cell annotation (middle plot). The right-most plot shows partition-based graph abstraction (PAGA) representation of the same data in left and middle plots, with nodes representing myeloid cell clusters and edges weighted based on the connectivity of different clusters. (F) Staining for *CD68*, *SPP1* and *HAVCR2* in biliary tumor tissue (three different patient samples are shown) and negative control (tonsil tissue). Examples of *CD68*$^{+}$*HAVCR2*$^{+}$*SPP1*$^{+}$ cells (orange arrowheads) are demarcated in overlay image.

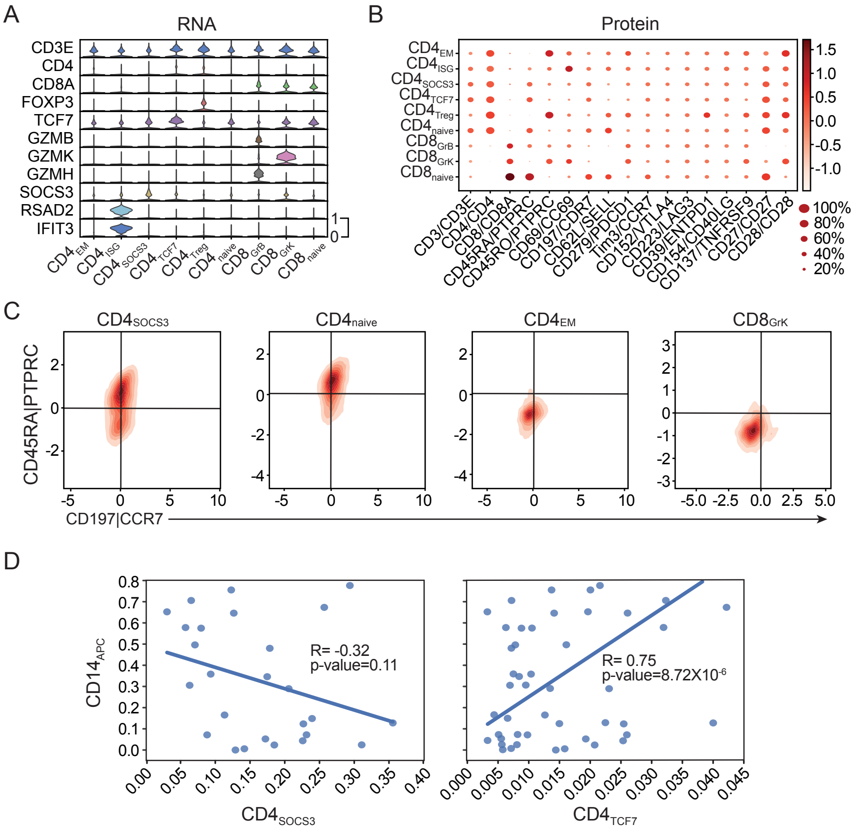

Figure S5. Peripheral T cell characterization and association with frequency of myeloid cell sub-types, related to Figure 5. (A) For each T cell type, mean expression of each gene is shown using a standard scale. (B) Expression of each protein is shown by percentage of cells with expression above the zero threshold (dot size) and mean expression (color) for each T cell type. (C) Protein expression of CD45RA (x-axis) and CCR7 (y-axis) for individual cells in the specified T cell types demonstrates examples of using protein data to annotate T cells as naïve, effector, and memory phenotypes. (D) The frequency of the specified cell type out of total myeloid or T cells was calculated and correlated as shown in each plot. Each dot corresponds to an individual patient sample.

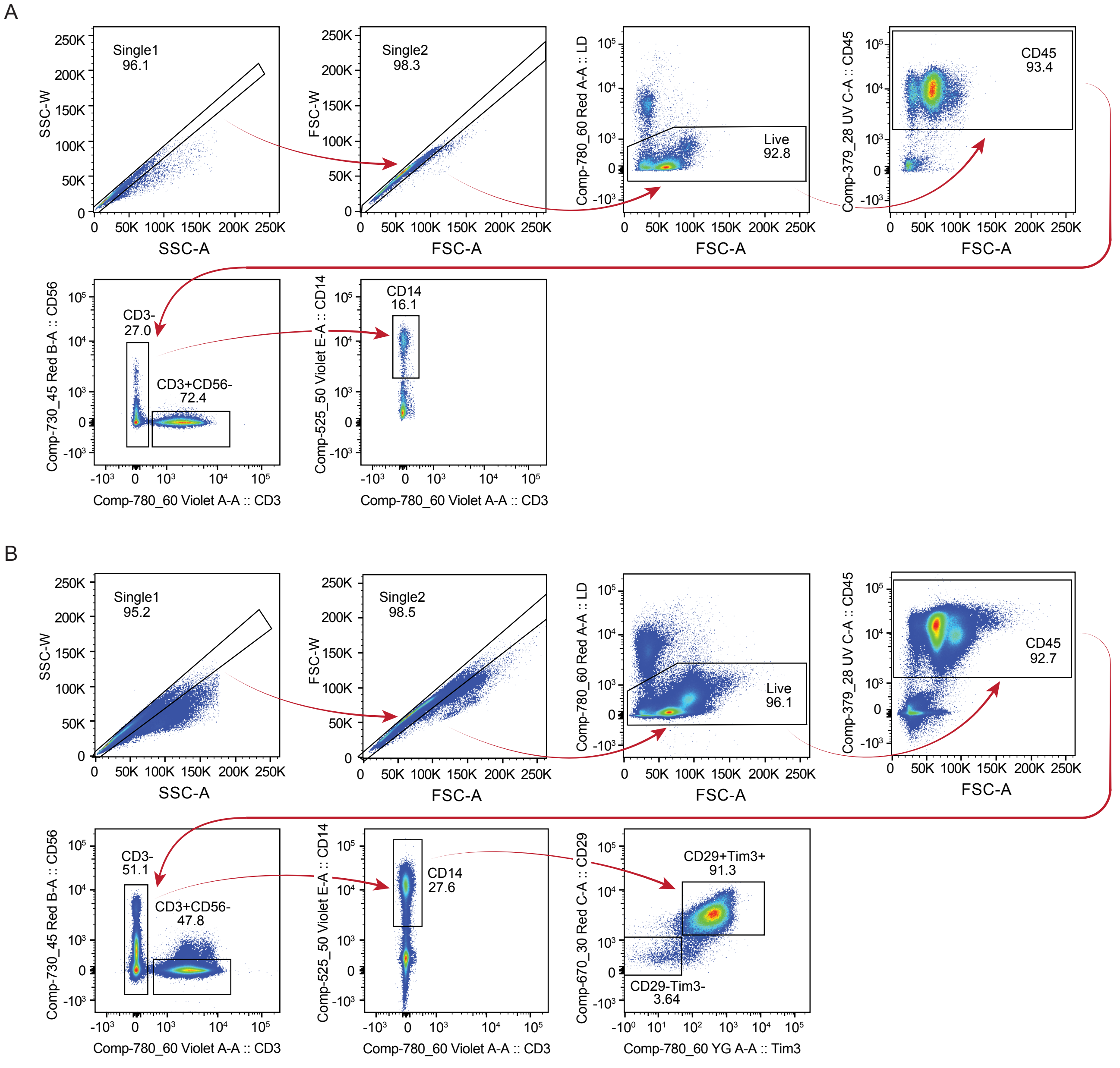

Figure S6. Fluorescence-assisted cell sorting for isolation and co-culture of monocyte sub-populations from healthy donors and BTC patients, related to Figure 5. (A-B) Sorting strategy for isolating myeloid cells from blood samples from healthy individuals (A) and BTC patients (B).

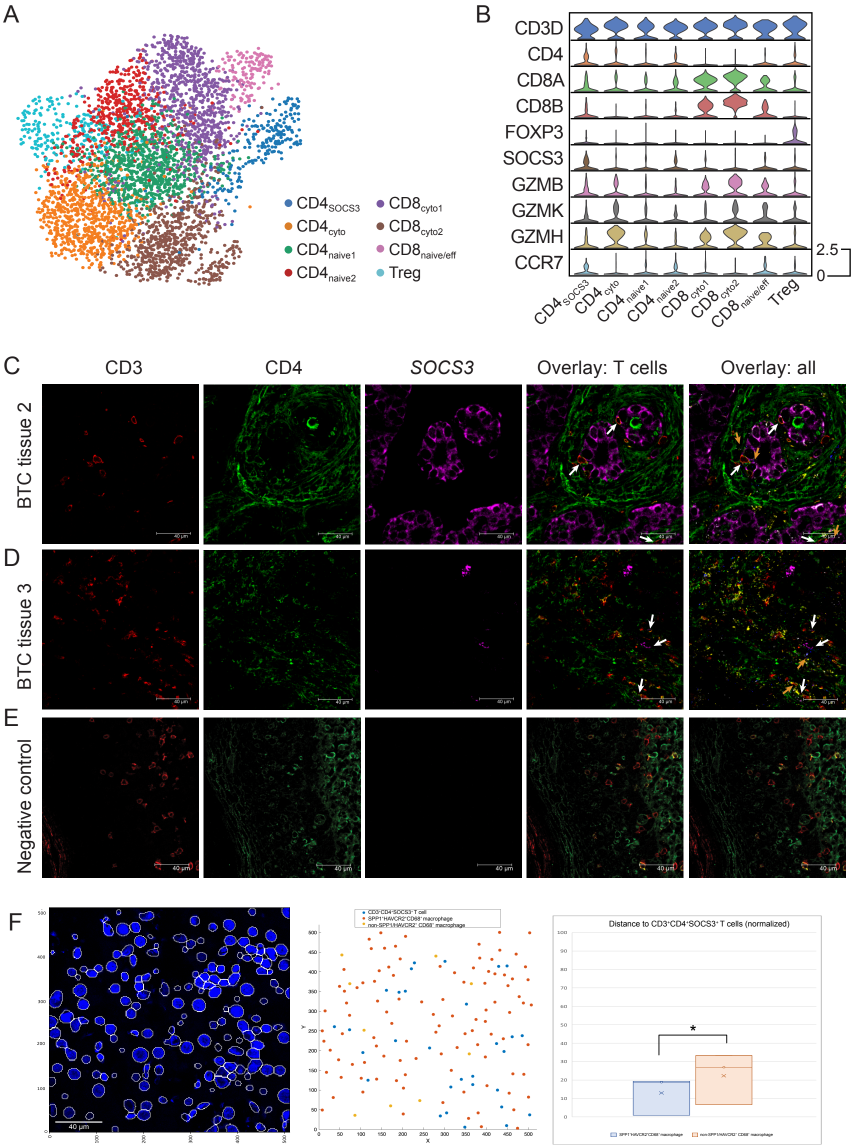

Figure S7. CD3^{+}CD4^{+}*SOCS3*$^{+}$ cells are identified in biliary tumors and co-localize with *CD68*$^{+}$*HAVCR2*$^{+}$*SPP1*$^{+}$ cells, related to Figure 5. (A) UMAP colored by cell annotations for intra-tumoral T cells. (B) For each intra-tumoral T cell type, mean expression of each gene is shown using a standard scale. (C-D) Staining for *SOCS3*, CD4, and CD3, is shown individually and overlaid (overlay: T cells), and with overlay of staining for *CD68*, *HAVCR2*, and *SPP1* (overlay: all) in representative biliary tumors (matched to BTC tumors shown in Figure 5 and S4). Examples of CD3^{+}CD4^{+}*SOCS3*$^{+}$ cells (white arrows) and of co-localization of CD3^{+}CD4^{+}*SOCS3*$^{+}$ cells with *CD68*$^{+}$*HAVCR2*$^{+}$*SPP1*$^{+}$ cells (orange arrows) are highlighted in overlay image. (E) Negative control staining for *SOCS3* probe with immunofluorescence for CD3 and CD4 in a control tonsil tissue. (F) Cell segmentation based on DAPI (nuclear) stain (left panel); cell centroids colored by cell types gated from segmentation output (single-cell arcsinh-transformed marker expression intensities) (middle panel); median distances of *SPP1*$^{+}$*HAVCR2*$^{+}$ and non-*SPP1*/*HAVCR2*$^{+}$ *CD68*$^{+}$ macrophages to CD3^{+}CD4^{+}*SOCS3*$^{+}$ T cells in images (n=3) from BTC tissues 1-3 (right panel, one-sided paired t-test, p=0.04). For left and middle panels, analysis from BTC tissue #1 was shown as an example.

Table S1. Patient and healthy donor clinical characteristics, related to Figures 1-5.

ID	Age	Gender	Race	Ethnicity	Cancer stage at diagnosis	Tumor type	Viral etiology	Clinical responder
BTC-1	54	M	White	Non-Hispanic/ Latinx	Stage IVB	Intrahepatic cholangiocarcinoma	No	No
BTC-2	65	M	Asian	Non-Hispanic/ Latinx	Stage IVB	Intrahepatic cholangiocarcinoma	HBV+ (core antibody positive, surface antigen negative)	Yes
BTC-3	58	F	White	Non-Hispanic/ Latinx	Stage IVB	Intrahepatic cholangiocarcinoma	No	Yes
BTC-4	68	F	White	Non-Hispanic/ Latinx	Stage IVB	Intrahepatic cholangiocarcinoma	No	No
BTC-5	62	M	White	Non-Hispanic/ Latinx	Stage IVB	Intrahepatic cholangiocarcinoma	No	No
BTC-6	53	F	Asian	Non-Hispanic/ Latinx	Stage III	Extrahepatic cholangiocarcinoma	No	Yes
BTC-7	66	F	White	Non-Hispanic/ Latinx	Stage IVB	Intrahepatic cholangiocarcinoma	No	Yes
BTC-8	61	F	White	Non-Hispanic/ Latinx	Stage II	Intrahepatic cholangiocarcinoma	No	No
BTC-9	73	M	Asian	Non-Hispanic/ Latinx	Stage IVB	Extrahepatic cholangiocarcinoma	HBV+ (core antibody positive, surface antigen negative)	No

HD-1	68	M	White	Non-Hispanic/Latinx	N/A	N/A	N/A	N/A
HD-2	71	F	Asian	Non-Hispanic/Latinx	N/A	N/A	N/A	N/A
HD-3	77	M	White	Non-Hispanic/Latinx	N/A	N/A	N/A	N/A
HD-4	46	M	Asian	Non-Hispanic/Latinx	N/A	N/A	N/A	N/A
HD-5	50	F	Asian	Non-Hispanic/Latinx	N/A	N/A	N/A	N/A
HD-6	50	F	White	Non-Hispanic/Latinx	N/A	N/A	N/A	N/A
HD-7	57	M	Asian	Non-Hispanic/Latinx	N/A	N/A	N/A	N/A
HD-8	50	F	White	Non-Hispanic/Latinx	N/A	N/A	N/A	N/A

Table S2. Antibodies and clones used in CITE-seq, related to Figures 1, 2, and 4.

**Antibody**	**Clone**
B7-H4	MIH43
CD1a	HI149
CD1c	F10/21A3
CD10	HI10a
CD103	Ber-ACT8
CD117	YB5.B8
CD11a	HI111
CD11b	M1/70
CD11c	B-ly6
CD123	7G3
CD124	hIL4R-M57
CD126	M5
CD127	HIL-7R-M21
CD13	WM15
CD133	W6B3C1
CD134	ACT35
CD137	4B4-1
CD14	MϕP9
CD141	1A4
CD152 (CTLA4)	BNI3
CD154 (CD40L)	TRAP1
CD155	TX24
CD16	3G8
CD163	GHI/61
CD178	NOK-1
CD18	6.7
CD183 (CXCR3)	1C6/CXCR3
CD184	12G5
CD19	SJ25C1
CD194 (CCR4)	1G1
CD195 (CCR5)	2D7/CCR5
CD196 (CCR6)	11A9
CD197 (CCR7)	3D12
CD2	RPA-2.10
CD20	2H7
CD206	19.2
CD21	B-ly4
CD226	DX11
CD235a	GA-R2 (HIR2)
CD24	ML5
CD25 (IL-2R)	2A3

CD26	M-A261
CD27	M-T271
CD270	CW10
CD272	J168-540
CD273	MIH18
CD274 (PD1L)	MIH1
CD275 (ICOSL)	2D3/B7-H2
CD278 (ICOS)	DX29
CD279 (PD-1)	EH12.1
CD28	CD28.2
CD29	MAR4
CD3	SK7
CD30	BerH8
CD314	1D11
CD32	FLI8.26
CD326	EBA-1
CD33	WM53
CD335 (NKp46)	9E2/NKp46
CD34	581
CD38	HIT2
CD39	TU66
CD4	SK3
CD40	5C3
CD44	G44-26
CD45	HI30
CD45RA	HI100
CD45RO	UCHL1
CD49a	SR84
CD49b	12F1
CD49d	9F10
CD49e	IIA1
CD5	UCHT2
CD54	HA58
CD56	NCAM16.2
CD61	VI-PL2
CD62L	DREG-56
CD69	FN50
CD7	M-T701
CD8	RPA-T8
CD80	L307.4
CD81	JS-81
CD86	2331 (FUN-1)
CD9	M-L13

CD90	5E10
CD94	HP-3D9
CD95	DX2
CD98	UM7F8
CXCR5 (CD185)	RF8B2
GITR (CD357)	V27-580
HLA-ABC	G46-2.6
IgD	IA6-2
IgG	G18-145
IL21R (CD360)	17A12
LAG3	T47-530
NKp44 (CD336)	p44-8
TCRab	IP26
TCRgd	B1
TIM-3	7D3

Table S3. P-values and fold change for immune cell frequency comparisons, related to Figures 1-3.

Comparison: Healthy donor versus Biliary tract cancer (BTC) patients pre-treatment, all immune cells

Cell type	Fold change	pvalue	Adjusted pvalue
B cell	-0.03011	0.151701	0.2085889
CD4+ T cell	0.016203	0.805537	0.881293
CD8+ T cell	-0.13046	0.004156	0.037686
NK	-0.02485	0.140799	0.2085889
NK/NKT	0.047816	0.088737	0.1626845
cDC	0.006557	0.024243	0.0630146
CD14+ mono	0.113645	0.028643	0.0630146
CD16+ mono	0.001102	0.881293	0.881293
pDC	0.003942	0.018931	0.0630146
plasma cell	-0.000891	0.006852	0.037686
progenitor	-0.002954	0.303424	0.3708516

Comparison: BTC responders versus non-responders pre-treatment, all immune cells

Cell type	Fold change	pvalue	Adjusted pvalue
B cell	-0.029232	0.488881	0.6722114
CD14+ mono	-0.009724	0.946072	0.988761
CD16+ mono	-0.000291	0.988761	0.988761
CD4+ T cell	0.035257	0.831595	0.988761
CD8+ T cell	-0.049857	0.097411	0.2678802
NK	-0.055735	0.071977	0.2639157
NK/NKT	0.105051	0.1651	0.36322
cDC	0.005143	0.488069	0.6722114
pDC	-0.004237	0.258185	0.4733392
plasma cell	-0.00084	0.040534	0.222937
progenitor	0.004464	0.003466	0.038126

Comparison: BTC responders versus non-responders at 1 week, all immune cells

Cell type	Fold change	pvalue	Adjusted pvalue
B cell	-0.065438	0.205547	0.7536723

CD14+ mono	-0.006977	0.926735	0.926735
CD16+ mono	0.007326	0.534267	0.926735
CD4+ T cell	0.066213	0.615903	0.926735
CD8+ T cell	-0.00677	0.858393	0.926735
NK	-0.005245	0.910505	0.926735
NK/NKT	0.007942	0.345301	0.926735
cDC	0.007132	0.043644	0.480084
pDC	-0.005103	0.094657	0.5206135
plasma cell	0.000143	0.818514	0.926735
progenitor	0.000777	0.837115	0.926735

Comparison: BTC responders versus non-responders at 3 weeks, all immune cells

Cell type	Fold change	pvalue	Adjusted pvalue
B cell	-0.082013	0.078001	0.2860037
CD14+ mono	0.233162	0.013425	0.147675
CD16+ mono	0.025849	0.039419	0.2168045
CD4+ T cell	-0.084377	0.355565	0.4763022
CD8+ T cell	-0.044454	0.157034	0.3454748
NK	-0.051041	0.199473	0.3657005
NK/NKT	0.000884	0.258632	0.4064217
cDC	0.000672	0.846558	0.846558
pDC	-0.002906	0.128991	0.3454748
plasma cell	-0.000404	0.433002	0.4763022
progenitor	0.004629	0.404162	0.4763022

Comparison: Healthy donor versus BTC patients pre-treatment, myeloid cells

Cell type	Fold change	pvalue	Adjusted pvalue
CD14_CTX	0.106337	0.19844	0.277816
CD14_ISG	0.050707	0.513194	0.5987263
CD14_IFL	-0.503573	0.000003	0.000021
CD14_APC	0.430743	0.006111	0.0213885
CD16_mono	-0.062957	0.044416	0.077728

cDC	-0.01844	0.038108	0.077728
pDC	-0.002817	0.852105	0.852105

Comparison: BTC responders versus non-responders pre-treatment, myeloid cells

Cell type	Fold change	pvalue	Adjusted pvalue
CD14_CTX	-0.085725	0.592656	0.963311
CD14_ISG	0.115711	0.446594	0.963311
CD14_IFL	-0.057482	0.001078	0.007546
CD14_APC	0.04452	0.836008	0.963311
CD16_mono	-0.001934	0.963311	0.963311
cDC	-0.001074	0.929414	0.963311
pDC	-0.014015	0.61744	0.963311

Comparison: BTC responders versus non-responders at 1 week, myeloid cells

Cell type	Fold change	pvalue	Adjusted pvalue
CD14_CTX	-0.056282	0.675966	0.788627
CD14_ISG	0.000916	0.632967	0.788627
CD14_IFL	0.049178	0.113661	0.656264
CD14_APC	-0.032417	0.796287	0.796287
CD16_mono	0.03109	0.426038	0.788627
cDC	0.033795	0.187504	0.656264
pDC	-0.02628	0.459307	0.788627

Comparison: BTC responders versus non-responders at 3 weeks, myeloid cells

Cell type	Fold change	pvalue	Adjusted pvalue
CD14_CTX	-0.493558	0.005812	0.01356133
CD14_ISG	-0.000874	0.289613	0.4054582
CD14_IFL	0.027247	0.649247	0.649247
CD14_APC	0.519527	0.001245	0.0043575
CD16_mono	0.021317	0.457643	0.53391683
cDC	-0.03142	0.016753	0.02931775
pDC	-0.042238	0.000128	0.000896

Table S7. Clinical characteristics of BTC resection samples, related to Figures 5, S4, and S7.

Sample number	Viral status	Prior treatment	Age (years)	Gender	Tumor classification
Tumor-1	non-viral	None	64	Male	Extrahepatic cholangiocarcinoma
Tumor-2	HBV cAb positive, HBV sAg negative, HBV sAb positive	None	43	Female	Intrahepatic cholangiocarcinoma
Tumor-3	non-viral	4 cycles gemcitabine /cisplatin, portal vein embolizatio n	60	Male	Intrahepatic cholangiocarcinoma
Tumor-4	non-viral	None	37	Male	Intrahepatic cholangiocarcinoma

HBV = hepatitis B virus, HCV = hepatitis C virus, cAb = core antibody
sAg = surface antigen, sAb = surface antibody
